# The Influence of Hydroxyapatite Crystals on the Viscoelastic Behavior of Poly(vinyl alcohol) Braid Systems

**DOI:** 10.3390/biomimetics9020093

**Published:** 2024-02-05

**Authors:** Tiago Quinaz, Tânia F. Freire, Andrea Olmos, Marcos Martins, Fernando B. N. Ferreira, Marcelo F. S. M. de Moura, Andrea Zille, Quyền Nguyễn, José Xavier, Nuno Dourado

**Affiliations:** 1 CMEMS-UMinho, Departamento de Engenharia Mecânica, Campus de Azurém, Universidade do Minho, 4804-533 Guimarães, Portugalnunodourado@dem.uminho.pt (N.D.); 2 INESC TEC, R. Dr. Roberto Frias, 4200-465 Porto, Portugal; 3 2C2T—Centro de Ciência e Tecnologia Têxtil, Departamento de Engenharia Têxtil, Campus de Azurém, Universidade do Minho, 4804-533 Guimarães, Portugalquyen@2c2t.uminho.pt (Q.N.); 4 Departamento de Engenharia Mecânica, Faculdade de Engenharia da Universidade do Porto, 4200-464 Porto, Portugal; mfmoura@fe.up.pt; 5 UNIDEMI, Department of Mechanical and Industrial Engineering, NOVA School of Science and Technology, Universidade NOVA de Lisboa, 2829-516 Caparica, Portugal; 6 LASI, Intelligent Systems Associate Laboratory, 4800-058 Guimarães, Portugal; 7 LABBELS—Laboratório Associado, 4710-057 Braga, Portugal

**Keywords:** poly(vinyl) alcohol, hydroxyapatite, braided composites, mechanical characterization, viscoelasticity, dynamic mechanical analysis

## Abstract

Composites of poly(vinyl alcohol) (PVA) in the shape of braids, in combination with crystals of hydroxyapatite (HAp), were analyzed to perceive the influence of this bioceramic on both the quasi-static and viscoelastic behavior under tensile loading. Analyses involving energy-dispersive X-ray spectroscopy (EDS) and scanning electron microscopy (SEM) allowed us to conclude that the production of a homogeneous layer of HAp on the braiding surface and the calcium/phosphate atomic ratio were comparable to those of natural bone. The maximum degradation temperature established by thermogravimetric analysis (TGA) showed a modest decrease with the addition of HAp. By adding HAp to PVA braids, an increase in the glass transition temperature (Tg) is noticed, as demonstrated by dynamic mechanical analysis (DMA) and differential thermal analysis (DTA). The PVA/HAp composite braids’ peaks were validated by Fourier transform infrared (FTIR) spectroscopy to be in good agreement with common PVA and HAp patterns. PVA/HAp braids, a solution often used in the textile industry, showed superior overall mechanical characteristics in monotonic tensile tests. Creep and relaxation testing showed that adding HAp to the eight and six-braided yarn architectures was beneficial. By exhibiting good mechanical performance and most likely increased biological qualities that accompany conventional care for bone applications in the fracture healing field, particularly multifragmentary ones, these arrangements can be applied as a fibrous fixation system.

## 1. Introduction

According to global figures, the amount of money allocated to fracture fixation devices ascended to USD 3.7 billion in 2007 [1]. Based on records of the American Academy of Orthopedic Surgeons (AAOS), each year, there are more than 6.3 million bone fractures reported in the United States, with very high expenditures [2,3] and considerable morbidity and mortality. Clinical challenges, such as the occurrence of delayed unions/non-unions and plate breakage, persist in the present day, with studies finding the prevalence of concerns in all fractures ranges from 3.5% to 13.3% [3,4]. A total of 5–10% delayed union and/or non-union, as well as diaphyseal fractures, can be observed, resulting in poor bone repair [5,6]. Many of these surgical procedures [7,8] demand the use of bone grafts and substitutes, particularly for serious multifragmentary diaphyseal fractures or considerable bone loss in long bones [8,9,10].

Osteosynthesis with plates and screws does not serve as a permanent substitute for fragmented bone [11], as these materials do not undergo degradation within the live organism [12]. Hence, the drawback associated with this provisional fixing method is the requirement for an extra procedure to eliminate metallic artifacts subsequent to the completion of fracture consolidation. The aforementioned issue carries significant societal and economic ramifications, presenting potential difficulties and yielding uncertain clinical outcomes [13,14]. Complications that may arise subsequent to the removal of the artifact encompass tissue and nerve damage, postoperative hemorrhage [13], infections, and refractures [8,10,13,15]. In addition, it should be noted that the metallic materials and mechanical devices currently applied in this context present certain limitations. One such limitation is their lack of biodegradability [12], which hinders their ability to naturally break down over time. Furthermore, these materials and devices often form weak connections with the surrounding native tissues [1], thereby impeding the establishment of robust integration. Additionally, their presence can elicit foreign-body tissue responses, such as necrosis following implantation [16], thereby jeopardizing the long-term functionality of the implant and the health of adjacent tissues [16].

The presence of an urgent clinical demand for treatments aimed at promoting rapid fracture repair and ensuring effective bone union in cases of long-bone multifragmentary diaphyseal fractures has been shown. The projected rise in prevalence and the substantial expenses linked to existing therapies will impose a significant financial strain on the healthcare sector in the forthcoming years.

Currently, there is a significant increase in the significance of 3D braided textiles in the fields of reinforced composites as well as structural and medicinal applications [17,18]. Prior studies have employed composite structures consisting of braided formations of PVA and hydroxyapatite (HAp) in conjunction with porous polymeric materials. These composite structures have been deemed to possess satisfactory stability and safety, as indicated by previous research [19]. Polyvinyl alcohol (PVA) is a polymer that has significant promise in the field of biomedical engineering, as evidenced by its extensive application potential [5,20,21,22,23]. Moreover, PVA is gaining increasing global attention for its utilization in the production of biodegradable composites [23]. The utilization of polyvinyl alcohol (PVA) in the advancement of fibrous constructions, such as braids, for the purpose of ensuring mechanical stabilization of broken bone regions represents a highly promising research area with significant clinical and societal implications. Porous implants exhibit significant promise as possible applications for PVA-braided topologies. The utilization of fibrous arrangements in conjunction with natural bodily fluids and bone surfaces enables the exploitation of a large contact area, hence facilitating the ingrowth of bone cells and promoting the durability of the implant over an extended period of time [24]. Hence, the beneficial process of spontaneous repair of damaged and impaired bones is achieved [25]. A prior investigation conducted on PVA braids [26] has similarly exhibited their favorable thermal, mechanical, and viscoelastic characteristics when subjected to tensile loading.

Nevertheless, the use of pristine polyvinyl alcohol (PVA) as an implant material is hindered by its inability to achieve rapid, robust, and suitable adhesion while ensuring the necessary interfacial bonding strength with natural tissue. Therefore, the utilization of fixing methods may be necessary to boost its performance [27,28], as well as improve its ability to promote bone growth and integration (bioactive behavior) in orthopedic applications [29,30].

Hydroxyapatite (HAp) is a mineral composed of calcium phosphate [31,32], which is naturally present in enamel and bone [12,25,32,33,34,35]. Hydroxyapatite (HAp) has been extensively researched in several biological and therapeutic contexts [28,34,36] owing to its exceptional biocompatibility with bone, muscles, and skin, as well as its similarity to the natural bone mineral phase [14,21,29,30,35,37,38,39]. The material in question is a bioactive, non-metallic substance commonly utilized within the orthopedic field [1,12,32]. Its primary application involves filling voids in substantial bone defects [6,12,28,37,40], hence augmenting the volume and osteoinductive properties of autologous bone grafts for the purpose of repairing bone defects [41].

In recent times, there has been a growing use of this substance as a surface covering in various clinical implant materials [1,12,14,25,32,40,42]. These materials include both metals and, more recently, polymers [1,12,32]. The primary objective behind this application is to augment the ingrowth of bone tissue and promote the attachment of the implant to the host bone [1,14,32,34]. Therefore, the apatite layer composed of hydroxyapatite (HAp) serves to enhance osseointegration [12,16], which refers to the establishment of a direct and close connection between the implant and bone, without the presence of any disruptive fibrous tissue at the interface [16]. This phenomenon facilitates the expedited healing of bone tissue [19,24,35]. In addition, it should be noted that hydroxyapatite (HAp) exhibits a considerable degree of stability [43]. Moreover, biomaterials based on HAp demonstrate a gradual resorption rate within the living organism [14,44,45,46], emulating the natural bone tissue’s remodeling process that occurs in proximity to the implant [44]. However, it should be noted that hydroxyapatite (HAp) is classified as a ceramic material, which inherently exhibits a brittle nature [14,31,37,38,47,48]. Consequently, its utilization as a standalone material is restricted [30,38,47,48], particularly in load-bearing scenarios [21].

To overcome the mechanical stiffness limitation of PVA filaments [22,33], HAp, a bioceramic, is commonly utilized in conjunction with polymers, such as PVA, to fabricate composite structures and scaffolds that possess enhanced mechanical characteristics [19,31,34,45,48]. Additionally, these possess the ability to manipulate the geometry of a polymer [34,49], exhibit resistance to degradation, demonstrate biological affinity [34,45], and enhance bioactivity in the context of osseointegration [19,24,30]. As a result, each component of the composite system is utilized [45], resulting in a biomedical composite with desirable properties.

Incorporating HAp into PVA filaments allows for enhanced mechanical compatibility and interfacial bonding strength of the resulting PVA/HAp composites with living skeletal tissues, among other natural tissues. Therefore, the artificial implant exhibits enhanced adhesion properties in general, which facilitates improved adhesion and proliferation of osteoblasts within the implant. This, in turn, promotes the formation of interfacial bioactive bonds with natural human bone [27,30]. Moreover, the integration of hydroxyapatite crystals (HAp) into PVA braids enhances their biocompatibility. Additionally, in vivo studies have demonstrated that HAp promotes increased regeneration and recuperation of bone tissue [19]. PVA/HAp braids in the form of a bone repair and immobilization system allow a uniform distribution of stress over the damaged region of bone (traumatic zones), are far less intrusive to the vascular system of bone tissue than other biocomposites, allow gradual drug release (pharmaceutical charges) and bioceramic (HAp), and potentially, do not require surgical revision procedures for revision (due to their natural biodegradable nature). They also have great potential when it comes to customization (the shape of the immobilization system).

Limited research has been dedicated to examining the viscoelastic properties of PVA fibrous architectures alone, the effect of HAp deposition upon creep and relaxation (which are fundamental viscoelasticity characteristics), or the assessment of frequency- and temperature-dependent properties of these biomaterials through DMA submitted to tensile loading. In addition, comprehensive knowledge regarding thermal degradations via DTA and TGA is critical for the finalization of their thermal characterization. Therefore, the purpose of this paper is to address this deficiency in the literature by detailing the investigation into the improved mechanical properties of poly(vinyl) alcohol braided yarns (PVA BY) reinforced with HAp. Multiple characterization techniques, such as SEM, EDS, and FTIR spectroscopy, provided substantial support for the findings. In addition, a range of mechanical tensile property tests were conducted alongside creep and relaxation experiments to assess the viscoelastic properties. Additionally, DMA was refined using thermoanalytical techniques. By contrasting the outcomes of the experimental group (PVA/HAp braids) and the control group (neat PVA braids), this study generates novel insights and forecasts for the future.

## 2. Materials and Methods

### 2.1. Materials

**Figure 1:**
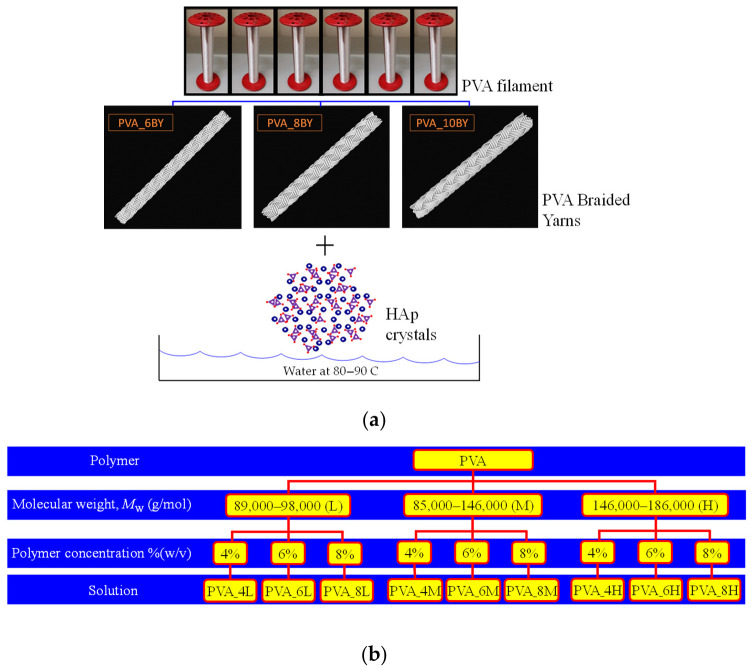
Representation of (**a**) the fabrication of various fibrous structures and (**b**) the analyzed and evaluated PVA solutions.

The PVA six-filament yarn (Mintval^®^, Besana In Brianza, Italy) utilized in this investigation was acquired by the melt spinning process of Exceval^®^ polymer from Kuraray^®^ (Osaka, Japan). Poly(vinyl alcohol) multifibre/multifilament yarns, exhibiting 41 dtex (36.9 denier; average fiber diameter: 26.10 μm), dissolved in water at 80–90 °C, were utilized in the fabrication of various fibrous structures (Figure 1a), such as biaxial braided ones. PVA powder 99+% (*M*_w_ = 89,000–98,000), PVA powder 87–89% (*M*_w_ = 85,000–146,000), and PVA powder 99+% (*M*_w_ = 146,000–186,000), all hydrolyzed, were acquired through Sigma Aldrich (USA). The molecular weight of hydroxyapatite (HAp, Sigma, USA) is 502.31 g/mol. The remaining reagents were of analytical quality and were employed without any additional refinement. They were acquired from Sigma-Aldrich in St. Louis, MO, USA.

### 2.2. Preparation of PVA/HAp Composite Braids

The PVA solutions and subsequent PVA/HAp mixtures were prepared in a manner consistent with that of prior research [19,35,50,51].

In the initial stages, nine distinct PVA solutions were prepared (Figure 1b), each containing a different concentration and type of PVA powder: (4L, 4M, 4H, 6L, 6M, 6H, 8L, 8M, 8H). “L” represents PVA powder with a low molecular weight (*M*_w_ = 89,000–98,000), “M” represents PVA powder with a medium molecular weight (*M*_w_ = 85,000–146,000), and “H” represents PVA powder with a high molecular weight (*M*_w_ = 146,000–186,000).

The concentrations of the PVA in the solution are represented by the values of “4”, “6”, and “8” in % (*w*/*v*). PVA aqueous solutions, consisting of distilled water, were prepared in each case with a final volume of 60 mL. Upon completion, the solutions were subjected to constant agitation for a duration of 2 h at a stirring plate temperature of around 90–100 °C, with the objective of achieving solutions that were transparent and homogeneous. The nine PVA solutions resumed in Figure 1b were synthesized through the dissolution of the polymer in distilled water, employing various concentrations and molecular weights.

The addition of the HAp to form PVA/HAp blends constituted the second stage. The addition of HAp constituted the second stage in the formation of PVA/HAp mixtures. With the objective of achieving the desired ductility and biodegradability necessary to replicate the properties of natural bone, a few authors propose the use of small quantities of HAp [52]. Therefore, this study investigated the effects of HAp addition at three distinct concentrations (1%, 5%, and 10% (*w*/*v*) of HAp). By subjecting the mixtures to agitation at an approximate temperature of 150 °C for a duration of 2 h, uniform milky coloration solutions were produced. The literature has previously documented the presence of this milky coloration, which is associated with HAp formation [53]. Following this, the solutions were stirred consistently until they reached room temperature. Finally, three Petri dishes were used to transfer ten samples of each braided PVA architecture (6, 8, and 10 BY). These samples had undergone prior examination and processing, with one dish designated for each fibrous architecture. Subsequently, after undergoing five cycles of 10 s in the homogenizer at a frequency of 20,000 rpm, the PVA/HAp mixtures were carefully poured into Petri dishes until the PVA-braided structures were fully covered (dip-coating). The PVA-braided architectures were permeated by the PVA/HAp solutions as a result of the braiding’s hydrophobicity and permeability [37]. Neat PVA braids act as a matrix for HAp [18,52]. The aforementioned polymeric matrix facilitates osteoconduction and enables appropriate biodegradability and ductility to simulate bone performance. In addition, the Petri dishes were desiccated for one hour at an approximate temperature of 40 °C in an oven. Following that, the braids were individually extracted from the Petri dishes and, utilizing clothespins, stretched and suspended in the identical oven set at 40 °C for an additional hour. The resulting PVA/HAp composite braids were subsequently sealed inside containers to prevent airborne reactions or contamination. This procedure for testing and characterization enabled discussion and selection of the optimal PVA/HAp mixture. As a control measurement, pure PVA braids were also compared using the aforementioned procedure, with the exception of the second phase (HAp addition).

The proposed method for producing PVA/HAp composite braids has a number of advantageous characteristics, including a reproducible strategy, rapid and cost-effective production, and the absence of chemical precursors, including crosslinkers. Indeed, these structures are constructed from biocompatible and non-toxic materials, which led to the development of PVA/HAp composites that are completely environmentally benign [22]. Additionally, the absence of organic solvents constitutes even more merit.

### 2.3. Morphological, Structural, Thermal, and Mechanical Characterizations

#### 2.3.1. SEM and EDS Analyses

The analysis of fabrics was conducted employing an ultra-high-resolution field emission gun scanning electron microscope (FEG-SEM) system from NOVA 200 Nano SEM, FEI. A 10 kV acceleration voltage was selected in order to acquire secondary electron images. The surfaces of the samples were coated with a 25 nm film of Au-Pd (80–20 weight percent) using a sputter coater of high-resolution (208HR Cressington Company, which was fixed to an MTM-20 Cressington high-resolution thickness controller). An analysis was conducted on the morphology of the HAp layer that was applied to the PVA-braided architectures using the identical SEM technique. Subsequently, the elemental (atomic) compositions of the composite braids were analyzed, which included the Ca/P ratio of HAp. In order to accomplish this, the SEM apparatus utilized energy-dispersive X-ray spectroscopy (EDS) analysis with an EDAX Si (Li) detector and a 5 kV acceleration voltage.

#### 2.3.2. TGA

TGA and DTA analyses were performed to determine the thermal behavior of pure PVA braids and the influence of the addition of HAp to the PVA braids. The thermal analyses were conducted using an STA 7200 Hitachi^®^ (Fukuoka, Japan), which provides a simultaneous display of TGA and DTA. The thermal stability of PVA braids was studied in relation to the presence of HAp, revealing its effect. To this end, PVA/10%HAp composite braids and corresponding control groups (specimens) were subjected to a single heating step at 10 °C min^−1^ in a nitrogen atmosphere (200 mL min^−1^) using an aluminum pan. The heating stage spanned the temperature range of 25–500 °C. Prior to testing, the initial mass was determined using a digital Mettler Toledo calibration balance (AB 204-S/FACT Classic Plus, Greifensee, Switzerland). In addition to WL (in percentage) vs. temperature, derivative thermogravimetric (DTG) vs. temperature graphs were generated from TGA data. DTG analysis was conducted to accurately identify the maximum peaks associated with thermal transformation events. The temperature difference (∆T) between the sample and reference was plotted against the sample temperature (T) in the DTA data. For each case, the mass of dried residues was computed.

#### 2.3.3. FTIR Spectroscopy

The chemical bonds of HAp and functional groups, as well as potential structural variations and intermolecular interactions among the constituents of PVA/10%HAp-braids (denoted as 6, 8, and 10BY), were identified via FTIR spectroscopy. Therefore, an analysis of the modifications in the functional groups prior to and subsequent to the inclusion of the HAp was conducted by making use of a Shimadzu^®^ FTIR-8400S model IRAffinity-1 spectrometer (Shimadzu, Kyoto, Japan), equipped with a PIKE MIRacleTM single reflection ATR accessory that employed a crystal diamond/ZnSe (PIKE Technologies, Fitchburg, WI, USA). The registry of spectra was performed utilizing 45 images spanning the range of 400–4000 cm^−1^, with a resulting resolution of 4 cm^−1^. Each of the measurements was performed in triplicate.

#### 2.3.4. Tensile Tests

Uniaxial tensile tests were conducted in 6, 8, and 10 BY of neat PVA, as well as in the form of a combined solution with HAp powder at 10% (i.e., 10%HAp). For this reason, the former was designated as 6BY, 8BY, and 10BY, whereas the latter resulted in the designations PVA_6BY/10%HAp, PVA_8BY/10%HAp, and PVA_10BY/10%HAp. The objective of the monotonic tensile experiments was to determine the ultimate strain, Young’s modulus, and ultimate strength of the aforementioned structures. A minimum of ten specimens were evaluated for each architecture, including pure PVA and composites of PVA/10%HAp.

Monotonic tests were conducted employing testing equipment from Hounsfield Universal (H100KS), using a load cell with a capacity of 250 N, a velocity setting of 100 mm/min, and room temperature (≈21 °C). Precise specimens of each sample were fixed between the testing grips, resulting in a 100 mm gauge length.

#### 2.3.5. Creep Tensile Tests

Homemade apparatus was utilized to conduct creep tensile tests in order to identify the viscoelastic properties. A piece-wise function with magnitude *σ*^0^ was imposed for a period of 7200 s, with the acquisition frequency set to 5 Hz (tolerance: ±1%). The value of *σ*^0^ was set to 12% of the attained tensile strength *σ*^u^ (true stress) in the linear elastic regime for the control group (i.e., neat-PVA_6BY, neat-PVA_8BY, and neat-PVA_10BY) and experimental group (PVA_6BY/10%HAp, PVA_8BY/10%HAp, and PVA_10BY/10%HAp), respectively. Tensile measurements were conducted to determine the value of *σ*^u^ for each braided architecture. Ten specimens were evaluated per braided configuration.

#### 2.3.6. Relaxation Tests

Tensile loading relaxation tests were conducted on the equipment and braided configurations (6BY, 8BY, and 10BY) that were mentioned earlier. A piece-wise function with magnitude *ε*^0^ was considered for the same period (2700 s), with the frequency of acquisition set to 5 Hz (tolerance: ±1%). The value of *ε*^0^ was set equal to the one that had been obtained when stress reached the threshold of 12% of the *σ*^u^ (true value) in the linear elastic regime for the control group (neat-PVA_6BY, neat-PVA_8BY, and neat-PVA_10BY) and experimental group (PVA_6BY/10%HAp, PVA_8BY/10%HAp, and PVA_10BY/10%HAp), respectively. Ten specimens were also evaluated for braided configuration.

#### 2.3.7. DMA

The evaluation of the viscoelastic behavior of neat PVA and PVA/10% braids was performed through dynamic mechanical analysis (DMA) in tensile mode using a 7100 DMA model manufactured by Hitachi^®^ (Fukuoka, Japan). An adequate (inert) environment was maintained using nitrogen (200 mLmin^−1^) during the experiments. The frequency range of the programmed synthetic oscillation was 0.1 to 2 Hz. Storage and loss moduli (*E*’ and *E*″) were measured in the temperature range of 25–160 °C, as well as the corresponding tan*δ* (loss tangent), while establishing 3 °C min^−1^ as the heating rate, both for the neat and the PVA/10%HAp composite braids. The aforementioned data were directly computed by the operating software of the equipment.

## 3. Results and Discussion

### 3.1. Characterisation of PVA Solutions

As evidenced by the data in Figure 1b, it was noted that the PVA solutions containing the highest (H) *M*_w_ required a more extended period of time to completely dissolve in comparison to the PVA solutions containing the lowest (L) *M*_w_. The preparation of solutions containing PVA_4H, PVA_6H, and PVA_8H failed to result in a homogeneous solution due to incomplete dissolution of the polymer (the solutions became excessively saturated). Consistent with previous research, these results demonstrate that an increase in the molecular weight of PVA with an extensive level of hydrolysis results in a greater viscosity of the aqueous solution [54], which was achieved in the present study, while the concentration of the solution remains unchanged. As a result, the solutions exhibiting higher *M*_w_ (H) values were excluded from the subsequent experimental procedures. Only six PVA solutions remained, namely PVA_4L, PVA_4M, PVA_6L, PVA_6M, PVA_8L, and PVA_8M, which were utilized to produce the subsequent PVA/HAp mixture.

### 3.2. Selection of PVA/HAp Mixture

In the process of determining the optimal PVA/HAp mixture (solution) for the production of the final PVA/HAp composite braid, two variables were taken into account. The first one regarded the molecular weight of PVA powder and percentage (*w*/*v*). The testing process involved six distinct varieties that were deemed eligible: PVA_4L, PVA_4M, PVA_6L, PVA_6M, PVA_8L, and PVA_8M. The second variable was the percentage of HAp content that had been used (1%, 5%, or 10% (*w*/*v*)). A compromise was reached between the percentage of HAp incorporation and the mechanical behavior in this study (i.e., the adequate tensile resistance to perform the braiding operations). Furthermore, the volume percentage of HAp in the composite should be increased if a specific biomedical application, such as fracture recovery, necessitates a high degree of bioactivity. Therefore, it is possible to modify the bioactivity and mechanical properties of the composite filament by adjusting the amount of HAp [28]. Therefore, following morphological, chemical, thermal, and mechanical analyses, the optimal PVA/HAp ratio of “PVA_6L_10%HAp” was determined and utilized in the production of the ultimate PVA/HAp composite strands. M.Enayati et al. [22] determined the selected ratio using comparable criteria.

### 3.3. Experimental Tests of PVA/10%HAp Composite Braids and Pure PVA Braids

The experimental tests, both with and without the addition of HAp, were conducted under identical conditions and using identical braided architectures. The subsequent results of characterization were acquired with the selection of the most effective PVA/HAp braided composite (“PVA_6L_10%HAp”).

#### 3.3.1. SEM and EDS Analyses

**Figure 2:**
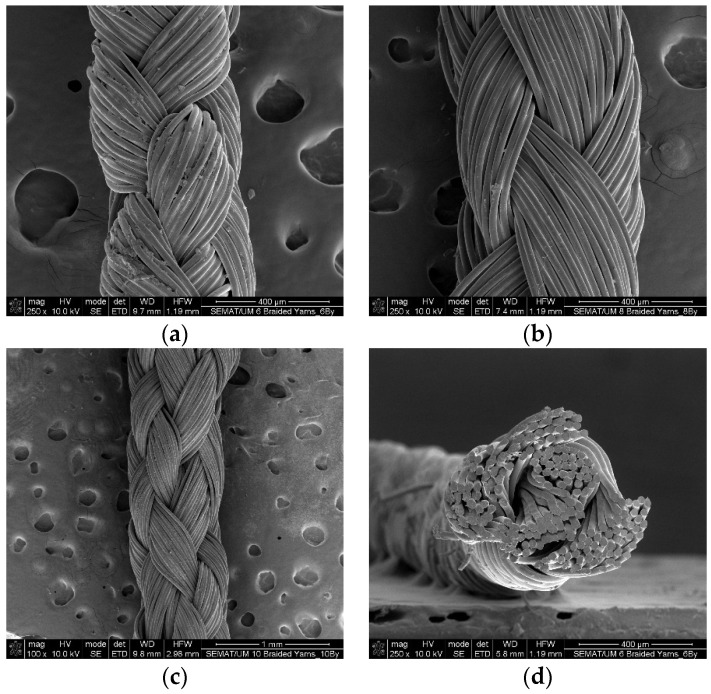

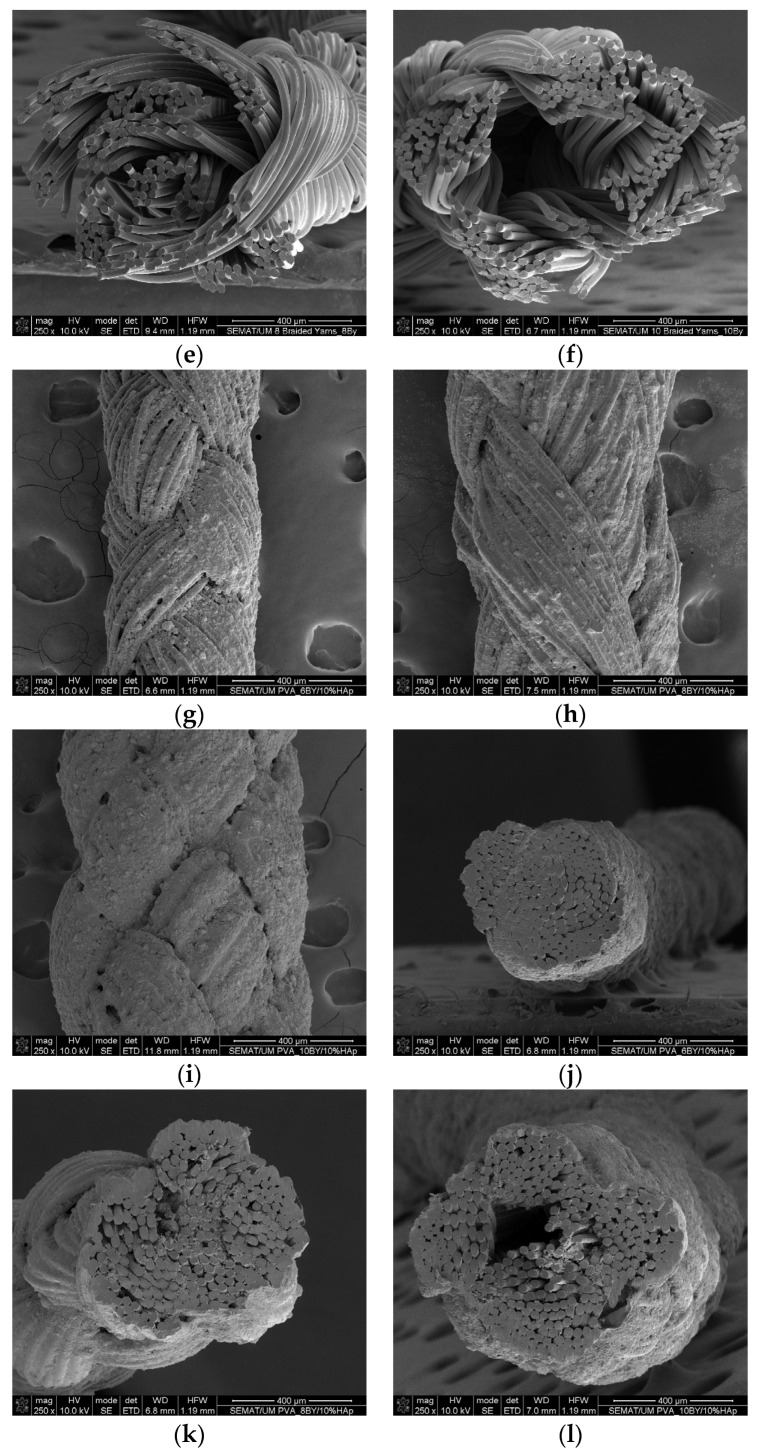
Micrographs by SEM of: (**a**) PVA_6BY front view; (**b**) PVA_8BY front view; (**c**) PVA_10BY front view; (**d**) PVA_6BY cross-section; (**e**) PVA_8BY cross-section; and (**f**) PVA_10BY cross-section; (**g**) PVA_6BY/10%HAp front view; (**h**) PVA_8BY/10%HAp front view; (**i**) PVA_10BY/10%HAp front view; (**j**) PVA_6BY/10%HAp cross-section; (**k**) PVA_8BY/10%HAp cross-section; and (**l**) PVA_10BY/10%HAp cross-section.

The front and top views of the SEM images of the examined PVA/10%HAp composite braided architectures are presented in Figure 2 (250× magnification). The corresponding neat PVA-braided architectures are available for reference [26]. The morphological distinctions between the orderly PVA structures mentioned in reference [26] (Figure 2a–f) and those containing (HAp) crystals are evident in Figure 2g–l.

Neat-PVA fibers/filaments (Figure 2a–f [26]) and PVA/10%HAp composite fibers’ mean diameters are comparable and have not changed substantially, as shown by SEM images (Figure 2g–l). Analogous findings were documented by Ruiz-Santos et al. [35]. A thin layer of HAp is visible in the center of the braids, in addition to a coating that completely covers the surface of the braids. Undoubtedly, a compact and consistent HAp coating layer is observed on the fiber surfaces, representing a dense and homogeneous spreading of HAp crystals. Notably absent are any substantial agglomerates, which undoubtedly contribute to the increased surface roughness of PVA braiding. A fibrous surface with greater roughness enhances surface wettability (i.e., hydrophilicity) [40,52], facilitates osseointegration (bone cell attachment) [24,25,40,52], stimulates cell proliferation and osteogenic differentiation [24,52], and improves the diffusion of nutrients [40]. Moreover, in the production of polymer-based composites, the mechanical properties of the structure are diminished due to the formation of agglomerates and poor interfacial strength [53]. Consequently, HAp must be distributed uniformly. In a study conducted by P. Yusong and X. Dangsheng [27], a homogeneous dispersion of HAp in the PVA matrix was attained with a low concentration of HAp. However, when a high concentration (content) of HAp was utilized, there was a propensity for HAp to aggregate within the polymer matrix, including PVA [27,45]. However, C. Lou et al. [27] suggested that HAp crystals aggregated into blocks could increase their surface area. Since the adhesion of HAp to the braid surface is strongly correlated with the proportion of HAp content [19], this demonstrated that HAp could be effectively impregnated into the braided architectures estimated in this study via the infusion of PVA/HAp mixtures.

However, the apertures that were present in the braided structures in the absence of HAp vanish when HAp is introduced; this is particularly apparent in the 10BY structure (Figure 2i). This occurrence can be attributed to the presence of the PVA/HAp mixture, which subsequently adheres to and permeates through the preexisting pores of the braided structures. The apertures are subsequently sealed subsequent to the mixture’s solidification [19]. The aforementioned occurrence results in the development of an HAp layer that is either more or less dense on the surface, depending on the configuration of the PVA/10%HAp composite and the associated pore dimensions. Therefore, the increased pore size observed in the 6BY/10%HAp structure results in a greater quantity of HAp particles penetrating into the core, leaving behind a thinner HAp layer on the surface. The opposite holds true for the 10BY/10%HAp composite system. The porosity of the composite structures that are produced is an essential property. Throughout their in vivo applications, it is imperative to ensure the efficient transfer of nutrients and the uninterrupted elimination of degradation products from the polymer [45].

Cross-sectional views (Figure 2j–l) illustrate that the structure of the neat PVA braids (illustrated in [26]) is designed with a looser structure, whereas the structure of the fibrous composite arrangement is much more compacted/closed, with the hollowness within the braids’ core diminishing as the distance between fibers decreases (the adhesion effect). As illustrated in Figure 2l, the PVA_10BY/10%HAp structure exhibits not only an increased quantity of fibers but also a number of fiber bundles that are dispersed across various regions and whose motion will vary in response to load application. This indicates that the force distribution in the 10BY system will be entirely different. The variations in the mechanical response of the braids can be elucidated by these observations in the presence of HAp. Comparable occurrences were detected by C. Lou et al. [55] as a result of glutaraldehyde crosslinking in PVA braiding. In comparison to untreated PVA braids, bone scaffolds consisting of PVA braids that were crosslinked using glutaraldehyde exhibited a much more compact structure, according to these authors.

**Figure 3:**
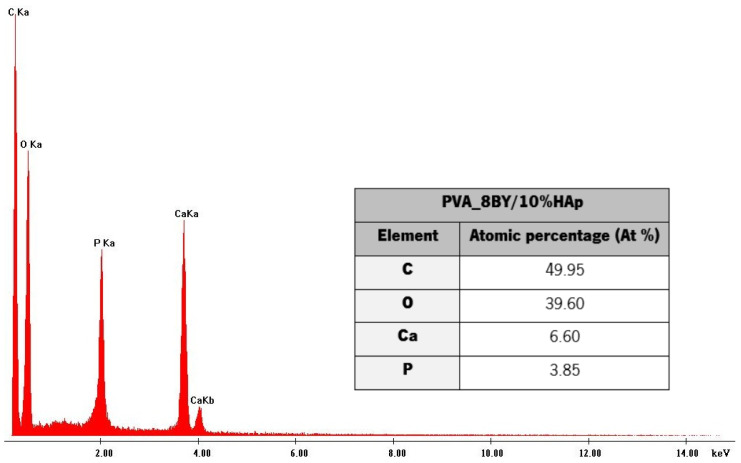
EDS spectrum determined on the PVA_8BY/10%HAp composite surface, with their chemical composition.

The fundamental composition of the HAp coating layer, as determined by EDS, is illustrated in Figure 3.

The main signals identified by EDS analysis (Figure 3) regard carbon (C), oxygen (O), calcium (Ca), and phosphorus (P), thus confirming that the PVA/10%HAp composite braids contain calcium phosphate (HAp). The Ca/P atomic ratio, which was estimated to be around 1.70 based on the EDS spectrum data, was determined. The Ca/P stoichiometric ratio of HAp observed in natural bone is comparable to this value (1.63 to 1.67) [12,18,34,36]. This confirms that the incorporation of HAp produces highly bioactive PVA-braided architectures [12]. An analogous Ca/P atomic ratio was determined by T. Li et al. [18] and the present study. The Ca/P molar ratio was estimated to be around 1.64 by P. Rodrigues and colleagues [36], while S. Uma Maheshwari et al. [30] reported a ratio of 1.62. The obtained EDS chars and chemical composition of the PVA_6BY/10%HAp and PVA_10BY/10%HAp structures were comparable to the ones that were presented. The phase purity of HAp crystals could have been confirmed by X-ray diffraction analysis (XRD), as it allows the analysis of amorphous and crystalline phases.

#### 3.3.2. TGA Measurements

**Figure 4:**
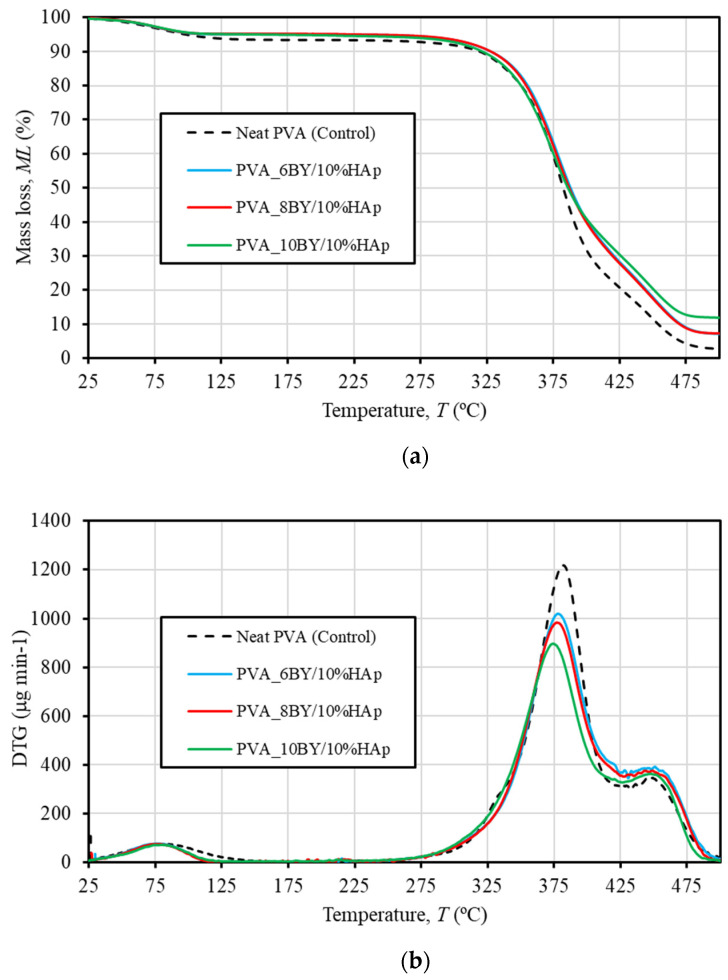
TG (**a**) and DTG (**b**) thermograms of neat −PVA braids (shown in dashed lines) and PVA/10%HAp composite braids (represented in continuous lines) in the range 25 to 500 °C.

TGA was used to examine the thermal decomposition (phase transitions) over time as a function of temperature to determine the thermal stability of neat PVA-braided systems (i.e., the control group) as well as the corresponding composites (the experimental group). The TGA and DTG thermograms of both pure PVA braids and PVA/10%HAp composite braiding are illustrated in Figure 4. The control group is represented by a single curve due to the high degree of similarity in behavior among the control architectures (neat-PVA_6BY, 8BY, and 10BY). The curve depicted in Figure 4a illustrates the weight loss (in percentage), denoted as ML (also in percentage), in relation to temperature *T* (between 25 and 500 °C). As depicted in Figure 4b, the DTG curve indicates the initial derivative of the TG over the same temperature interval.

The mean values of thermal properties pertaining to the three thermal peaks and the matching weight losses for every examined fibrous architecture are presented in Table 1. Therefore, it comprises the weight loss (WL) that took place during each phase of thermal degradation, in addition to the peak temperatures at which the degradation velocity (DTG peak) is at its greatest. Alternatively stated, DTG peaks indicate the location of the greatest mass reduction [49]. For each sample test, the mass of desiccated residues was additionally determined; this value was used to calculate the residual weight at 500 °C (wt%), as shown in Table 1.

As shown in Table 1 and the TG and DTG thermograms (Figure 4), the thermal degradation of every braided architecture occurs in three distinct thermal events or phases.

A first minor weight loss (WL) of around 5–6% of the total weight is detected until 125 °C. This WL is attributed to the evaporation of absorbed moisture or the confined and adsorbed water that has been absorbed [23,34,35,49,51,56,57]. This first decomposition procedure is composition-independent for every braided architecture under consideration. In addition, as shown in Table 1, the degradation of all fibrous architectures occurs in two main steps, each of which results in greater losses than the first. These steps are denoted as the second (approximately 250–425 °C) and third (425–500 °C) thermal degradations, respectively. This temperature range is referred to in the literature as being correlated with the structural degradation of PVA [23,34,35,49,51].

Table 1 provides the following DTG temperature values for the control group (illustrated by the dashed line in Figure 4): a peak at approximately 383 °C, which corresponds to 73% of WL, and a minor peak at around 447 °C, which corresponds to a value of 16% of WL. The dehydration reaction of PVA chains is attributed to the second and primary WL at 383 °C [56,57]. This reaction involves the side chain decomposition [22,49,56] and the corresponding formation of the polyene structure [56]. Conversely, the degradation of the polyene chain (carbonation) occurs around 447 °C and is represented by the smaller DTG peak and the third WL stage [34,51,56,57]. This corresponds to the main chain decomposition of PVA [29,49,56] and results in the production of carbon and hydrocarbons [57].

Consistent with the findings of R. Ruiz-Santos et al. [35], the outcomes perceived in the PVA/10%HAp composites demonstrate remarkably comparable TGA patterns. It is noteworthy that in Figure 4a, the contours associated with the PVA_6BY/10%HAp and PVA_8BY/10%HAp structures occur primarily concurrently. Furthermore, certain variations in thermal behavior become apparent when HAp is added. The velocity of water content loss, as indicated by the magnitude (intensity) of the initial peak of the DTG in Figure 4b and the quantity lost as WL (represented in percentage) documented in Table 1, exhibited distinct characteristics in fibrous structures composed of pure PVA and composite PVA/HAp. Therefore, it was deduced that the former structure (first thermal degradation) discharged marginally more water at a comparable rate than the latter. With respect to the second thermal degradation, the inclusion of HAp into the braids has no discernible impact on the onset temperature of PVA decomposition, as confirmed by TGA thermograms. The results presented here align with the research conducted by Enayati et al. [29]. The rate of degradation in the control group was considerably faster than in the experimental group (composite specimens), as indicated by the most intense DTG peak in Figure 4b. Nevertheless, the DTG thermograms (Figure 4b) and Table 1 indicate that the experimental group composites experienced a displacement of the second WL process towards a lower temperature region. Furthermore, the inclusion of HAp resulted in a marginal reduction in the maximum loss mass temperature (known as degradation), which is linked to the so-called degradation of PVA side chains. A. Asran et al. [49] documented this event as well. The observed result may be due to the robust interactions that exist between HAp and PVA, which include hydrogen bondings and/or hydroxyl-calcium [HO–]–Ca^2+^–[–OH] linkages [29,36]. Such interactions are hypothesized to weaken the side chains (hydroxyl groups) of PVA, thereby reducing the maximal loss mass temperature [20,29].

Additionally, it was noted that the sample containing neat PVA_10BY/10%HAp exhibited the lowest total WL of 86%, while the sample containing pure PVA_10BY exhibited the highest total WL of 96%. An additional noteworthy aspect is that, with respect to pure PVA braids, a weight loss of nearly 98% is maintained until the temperature of 500 °C, with a negligible carbonized residue content of approximately 2% (on average, as shown in Table 1). Conversely, at a temperature of 500 °C, the residual weight of PVA/10%HAp braids surpassed that of neat PVA braids and exhibited an upward trend as the quantity of PVA-braided fibers increased. Depending on the composite architecture, this accounted for an average of 7 to 12 wt% of the total weight (Table 1). Upon exceeding ≈ 480 °C, all TGA diagrams exhibited a flattened appearance. Given that the residual mass can be primarily attributed to the presence of inorganic residue (specifically the HAp mineral phase) [34,35,49], these results validate the addition of HAp crystals into the PVA braiding. Considering that the residual weight for the control group is estimated to be around 2%, it is feasible to deduce that the HAp concentration in the composites PVA_6BY/10%HAp and PVA_8BY/10%HAp was approximately 5% by weight. However, the PVA_10BY/10%HAp has the capability to disperse a significantly larger amount of HAp, specifically ≈ 10 wt%. The TGA data are supported by SEM observations, which indicate that the PVA_10BY/10%HAp composite architecture exhibited a higher HAp content that was readily noticeable on its surface subsequent to the addition of HAp. Therefore, the increased pore size exhibited by the PVA_6BY/10%HAp composite allows for a greater quantity of HAp particles to permeate into the core while leaving a thinner HAp layer on the surface; the PVA_10BY/10%HAp composite, conversely, demonstrates the opposite characteristic.

#### 3.3.3. DTA Measurements

**Figure 5:**
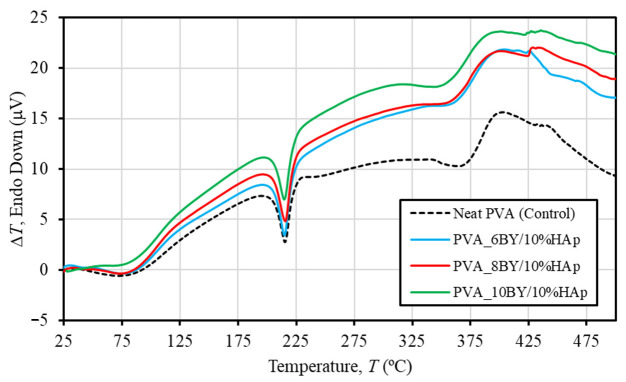
DTA curves of pure PVA braids are represented by dashed lines, and PVA/10%HAp composite braids are represented by continuous lines.

The DTA curves for pure PVA braids (control group) and PVA/10%HAp composite braids (experimental group) are depicted in Figure 5. In line with TGA/DTG analyses, the performance of the three control architectures (PVA_6BY, 8BY, and 10BY) demonstrated remarkable comparability. Consequently, a single curve is utilized to portray the control group. Furthermore, Table 2 provides an overview of the average thermal properties of the fibrous structures examined in this study, enabling a clearer differentiation and highlighting the impact of the incorporation of HAp on the thermal characteristics of PVA strands.

The DTA curves exhibited three distinct maxima across all braided architectures that were assessed (Figure 5). The shoulder peak in Figure 5 represents the glass transition temperature (*T*_g_) of PVA. The mean temperatures of neat-PVA_6BY, neat-PVA_8BY, and neat-PVA_10BY are given in Table 2. The average temperatures for PVA_6BY/10%HAp, PVA_8BY/10%HAp, and PVA_10BY/10%HAp are 68 °C, 72 °C, and 74 °C, respectively. These temperatures correspond to the amorphous portion of the material (*T*_g_) [56]. Therefore, the addition of HAp to PVA braids resulted in an increase in *T*_g_. The rise in quantity can be attributed to the strong hydrogen bond interactions between PVA and HAp, which limit the movement of the PVA chains along their segments [49]. Notwithstanding, the values of *T*_g_ that have been referenced must be verified during DMA. Furthermore, the strong second endothermic peak (*T*_m_) observed in both pure PVA structures and composites of PVA/10%HAp can be attributed to the PVA crystalline phase melting [49,56]. Table 2 displays the average temperatures for different samples. Neat PVA_6BY, neat PVA_8BY, and neat PVA_10BY exhibit average temperatures of 216 °C, 216 °C, and 215 °C, respectively. On the other hand, PVA_6BY/10%HAp, PVA_8BY/10%HAp, and PVA_10BY/10%HAp show an average temperature of 215 °C. As a result, the addition of HAp caused a negligible shift towards the lower value of *T*_m_, which may suggest that the crystallinity decreased as a result [22,30,49]. In their study, A. Asran et al. [49] similarly documented a diminished value when HAp nanorods were integrated into PVA nanofibres.

The observed decomposition of the residual acetate group [56], as well as the elimination of hydroxyl groups like water and the formation (synthesis) of polyene macromolecules [58], are correlated with the final and third peaks. The curves revealed in DTA for PVA/10%HAp composite braid exhibit a marginal displacement, suggesting a minor reduction in thermal stability due to the inclusion of HAp (Figure 5). The experimental group, consisting of PVA/10%HAp composite braids, exhibits a lower degradation temperature compared to the control group of pure PVA braids. This is evident from the third peak temperature depicted in Figure 5 and Table 2. The weakening of the PVA side chains leads to a decrease in the maximal temperature of bulk degradation, thereby facilitating this phenomenon [36].

#### 3.3.4. FTIR Spectroscopy Measurements

**Figure 6:**
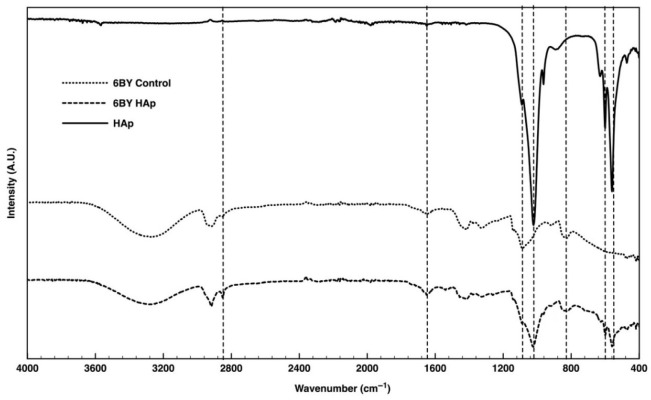
TR-FTIR spectra of neat-PVA and PVA/10%HAp braided architectures (6BY configuration).

The FTIR spectra of the pure PVA (serving as the control group) and PVA_6BY/10%HAp are presented in Figure 6. The results obtained for the PVA_8BY/10%HAp and PVA_10BY/10%HAp configurations demonstrate a significant level of similarity.

In the case of the pure PVA yarns (referred to as the “6BY Control”), the Fourier transform infrared (FTIR) data (Figure 6) revealed the following: methylene-CH2 deformation vibration peak at approximately 820 cm^−1^ [12], CH_2_-OH [25] peak at approximately 1085 cm^−1^ [45,50], C-O stretch shoulder within the interval 1093–1140 cm^−1^ [27,45,48], methyl group C-H stretching at around 1320 cm^−1^ [25], 1415 cm^−1^ [18,45], and 1650 cm^−1^ [25], C-H bending at 1440 [48], and C-H stretching [48,58] of the remaining methylene-CH_2_ and methyl-CH_3_. The peak observed at approximately 3280 cm^−1^ is indicative of the hydroxyl group’s (-OH) O-H stretching vibration [50,53,58].

Regarding the FITR pattern of purified HAp, each of the functional group-specific absorption bands of the HAp lattice was observed. The P-O bending vibration (ν4) of PO_4_^3−^ is associated with the double split bands at approximately 560 cm^−1^ and 600 cm^−1^. The P-O symmetric and asymmetric stretching modes (ν3) can be identified by the double split bands observed at around 962 cm^−1^ and 1040 cm^−1^, respectively [18,25,27,28,29,36,37,40,45,47,50,53]. The distinct low peak around 3550 cm^−1^, indicative of the stretching vibrations of the internal hydroxyl of HAp [12,18,25,48,49], is also evident.

The peak characteristics of the PVA/10%HAp composite braids closely matched those of the standard PVA and HAp patterns. Comparable to the spectrum of pure HAp, the Fourier transform infrared (FTIR) spectra of “6BY HAp” exhibit double split bands at approximately 560 cm^−1^ and 600 cm^−1^, which correspond to the P-O bending vibration (ν4) of PO_4_^3−^ ions, and at approximately 1024 cm^−1^ (shoulder peak), which signifies the P-O symmetric and asymmetric stretching vibrations (ν3) of the PO_4_^3−^ ion, respectively. These double split bands are distinctive features of the crystalline phosphate phase of HAp [18,22,25,27,28,36,37,40,45,47,53].

#### 3.3.5. Monotonic Tensile Tests

**Figure 7:**
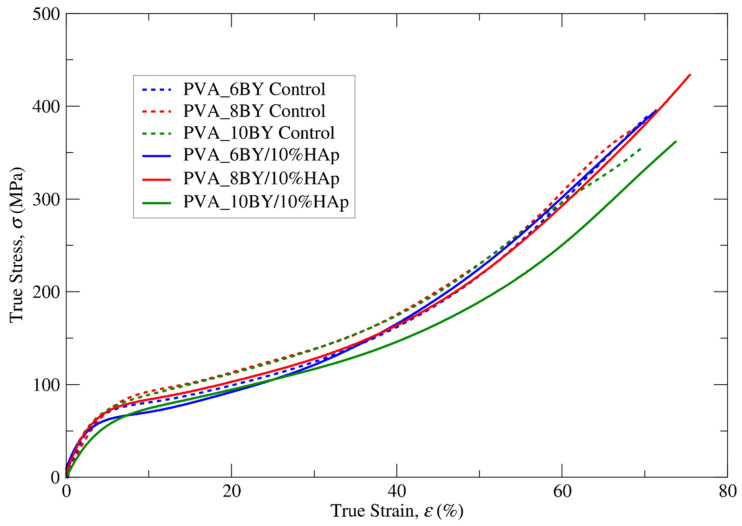
True tensile stress–strain mean curves for both neat PVA (control) and PVA/10%HAp braided architectures exhibit distinct characteristics, considering ten independent results per curve.

The true stress–strain curves for clean PVA braids and their PVA/10%HAp braided composites were obtained under tensile quasi-static loading conditions, as shown in Figure 7. In order to account for the reproducibility of the measurements (ten specimens per architecture), error bars are omitted from the averaged data plot. The mean true values of the tensile strength ($\sigma^{u}$), ultimate strain ($\varepsilon^{u}$), and Young modulus (*E*) derived for the structures under investigation are succinctly presented in Table 3. The aim of this table is to analyze the effect of incorporating HAp on mechanical properties.

The elastoplastic behavior of all designed braided specimens under tension is characterized by nonlinear kinematic hardening until the point of final rupture. This can be observed in Figure 7. This conduct bears resemblance to the findings documented by J. A. Cooper et al. [59] regarding alternative PLGA copolymer braiding structures. As reported by additional authors [29], PVA exhibits characteristics of a soft material, including low stiffness and comparatively high values of strain. Moreover, the addition of 10% (*w*/*v*) HAp is expected to result in a more brittle mechanical behavior as a consequence of the hard phase influence. However, this study did not observe this behavior.

A marginal modification in the nonlinear characteristics of the σ–ε curve was identified in the fibrous structures that were integrated with HAp (Figure 7 and Table 3), albeit one that was of lesser importance in the case of the PVA_6BY/10%HAp. The values of *E* (more flexible) exhibited a marginal reduction when HAp was incorporated into PVA braids (experimental group), as compared to neat PVA architectures (control group). The minimum value of *E* for PVA_10BY/10%HAp was observed to be 14.500 (16) MPa. S. Mollazadeh and colleagues [53] put forward the hypothesis that the composite groups exhibited lower mean values of *E* because of a more robust interface between the polymeric matrix and the filler particles. Furthermore, the microstructural components present in the PVA/10%HAp composite structures demonstrated a more unified response to the applied load, which further contributed to the observed decrease in *E*.

Based on the results presented in Table 3, it can be inferred that the presence of HAp (composite braids, experimental group) results in higher tensile strength and strain at rupture (represented by $\sigma^{u}$ and $\varepsilon^{u}$, respectively) in comparison to the absence of HAp (used as the control group). The tensile strength ($\sigma^{u}$) of the PVA_8BY/10%HAp composite was measured to be 433.392 (5) MPa, which was found to be higher than the tensile strength of other braided architectures. Specifically, it was around 8% higher than the tensile strength observed in the clean PVA_8BY architecture. In addition, the PVA_8BY/10%HAp composite system exhibited the highest value of $\varepsilon^{u}$, surpassing the value of the neat PVA-braided system (neat PVA_8BY) by at least 7%.

The interfacial adhesion between the polymeric matrix (PVA) and the filler particles (HAp) plays a crucial role in determining the mechanical properties of polymer matrix composites [53]. The SEM analyses confirmed the presence of a well-bonded and evenly dispersed microstructure of HAp on the region of the surface of the PVA braids. This suggests that there will be a more effective transfer of load and subsequent stress from the polymer matrix to the reinforced HAp crystallites. As a result, it was anticipated that the PVA/10%HAp composite braids would exhibit improved mechanical properties [23,53]. The confirmation of this hypothesis in our study highlights the significant contribution of the interaction between the PVA matrix and HAp particles in bolstering the tensile strength of the composite braids [57].

Modifications in the mechanical properties may imply the presence of chemical bonding between the HAp and PVA molecules, leading to evident consequences for its overall structure. The FTIR analysis provides evidence for these observations, as the vibration bands of each functional group in both PVA and HAp molecules remained present in the vibration bands of the PVA/10%HAp braided composites. The presence of the hydroxyl (-OH) group in the PVA molecule serves as an active site for the binding of calcium ions found in the HAp molecule [27]. This interaction can result in the creation of linkages involving [OH–]–Ca^2+^–[–OH] [22,34,49]. Furthermore, the alteration in the degree of tacticity and conformation of the PVA molecule occurred due to the abundance of -OH groups, resulting in the formation of a strong intermolecular hydrogen bond interaction between PVA and HAp [27,49].

The observed mechanical behavior can be attributed to the presence of HAp embedded in a PVA matrix and deposited on the surface of the braided structures. This combination of HAp and PVA acts as both a lubricant and reinforcement, resulting in an increase in both the ultimate strength ($\sigma^{u}$) and strain ($\varepsilon^{u}$). Therefore, these findings indicate that the inclusion of HAp enhances the mechanical properties of PVA/HAp composite braids, making them suitable for use in bone grafting and orthopedic implants [60].

#### 3.3.6. Creep Tensile Tests

**Figure 8:**
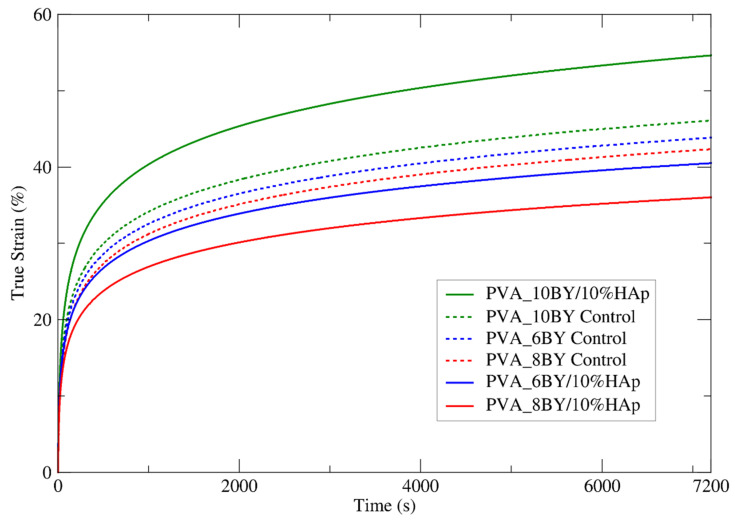
Experimental mean creep curves were obtained for both the neat PVA braids and their PVA/10%HAp composites over 7200 s.

The investigation involved conducting creep tensile experiments on two groups: the experimental group consisting of PVA_6BY/10%HAp, PVA_8BY/10%HAp, and PVA_10BY, and the control group consisting of neat PVA_6BY, PVA_8BY, and PVA_10BY. To ensure a uniform and abrupt increase in tensile stress, a fixed load was utilized. Consequently, for a period of 7200 s, input stress values ($\sigma_{0}$) of approximately 48.44 MPa, 49.65 MPa, 45.65 MPa, 48.60 MPa, 51.03 MPa, and 43 MPa (equivalent to 12% of the respective tensile strengths) were applied to the following materials: neat PVA_6BY, neat PVA_8BY, neat PVA_10BY, PVA_6BY/10%HAp, PVA_8BY/10%HAp, and PVA_10BY/10%HAp, respectively. Due to its significantly lower value compared to the ultimate tensile stress ($\sigma^{u}$) reached by the material under monotonic uniaxial tensile loading, $\sigma_{0}$ ensures a linear response throughout the entire loading procedure. In Figure 8, the mean creep curves of neat PVA braids and the corresponding PVA/10%HAp composites are shown. Additionally, Table 4 provides the mean true strain function for the stress input within a period of 7200 s. The figure uses mean creep curves from ten valid results (recovery from creep was not plotted).

A steady trend in the stress profile over time, which approaches a plateau, is illustrated in Figure 8. Certain discrete bundles of fibers undergo immediate straightening upon initial (i.e., instantaneous) stress, whereas others maintain their crimped state. The subsequent redistribution of stress across an increasing number of fibers (nonlinear recruitment) results in the straightening and loading of these fibers in a sequential manner, which aids in the reduction of creep. Creep is undoubtedly a phenomenon that exclusively takes place when fibers are in a straight alignment [61].

Based on the analysis of the mean curves for true tensile strain against time, as depicted in Figure 8 (a total of 10 samples per architecture), it can be concluded that the PVA_10BY/10%HAp composite exhibits a more deformable characteristic in comparison to the control sample. The strain values of the PVA_6BY/10%HAp and PVA_8BY/10%HAp systems were found to be lower in comparison to the control. The most deformable architecture was determined to be PVA_10BY/10%HAp, which stabilized at 55% creep strain. Furthermore, it exhibits the least disparity (18.7%) when compared to the final strain value reported in Table 4 ($\varepsilon^{u}$ = 73.7%). The least deformable system was PVA_8BY/10%HAp, which stabilized at 35% creep strain and exhibited the greatest deviation (40.6%) from the ultimate strain value (ᵢu = 75.558%). In addition, the 6BY structure has achieved 40% stability and demonstrates a substantial deviation of 31.210 percent from the ultimate strain ($\varepsilon^{u}$ = 71.210%).

The decrease in the percentage of deformation over time observed in the 8BY and 6BY composite architectures strongly suggests that the incorporation of HAp yielded beneficial results. The 10BY composite system eliminated the visibility of this effect. This may be the result of increased HAp impregnation in this structure (as indicated by the TGA data), which compromises its mechanical properties.

#### 3.3.7. Relaxation Tensile Tests

The control group (neat PVA_6BY, PVA_8BY, and PVA_10BY) and the experimental group (PVA_6BY/10%HAp, PVA_8BY/10%HAp, and PVA_10BY/10%HAp) were subjected to a sudden initial stress intensity similar to that observed in creep tensile tests within the linear elastic domain. This stress intensity was maintained for a duration of 2700 s. Therefore, the applied strain ($\varepsilon_{0}$) for the following composite architectures has been determined: 2.3% for neat PVA_6BY, 2.5% for neat PVA_8BY, 2.6% for 2.8% for PVA_10BY, PVA_6BY/10%HAp, PVA_8BY/10%HAp, and 3.3% for PVA_10BY/10%HAp. The values presented here were calculated by utilizing the strain value recorded by each braided structure at 12% of the tensile strength ($\sigma^{u}$).

**Figure 9:**
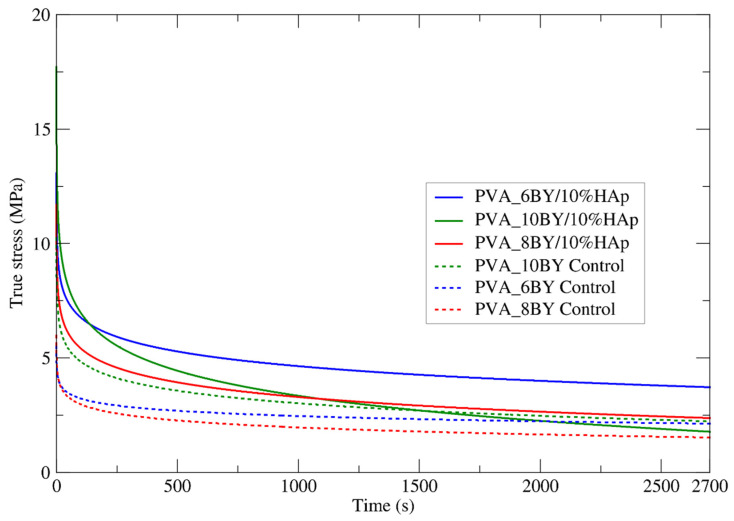
Experimental mean relaxation curves were obtained for both the neat PVA braids and their PVA/10%HAp composites, covering a time span of up to 2700 s.

To gain insights into the time-dependent properties of neat PVA braids and PVA/10%HAp composites, the average relaxation curves of these structures were carefully analyzed. The examination involved ten valid results, and the outcomes are illustrated in Figure 9. This analysis focused on the evolution of the attained true stress (*σ*) for each fibrous structure within a time interval of 2700 s (*t*) (Table 5).

Analogous to creep tensile experiments, the mechanical behavior of braided composites exhibits a consistent trend in this examination, culminating in a stress plateau.

The stress relaxation behavior, as stated by G. Thornton et al. [61], does not include the recruitment of fibers. During the application of the initial deformation, a limited number of fibers are recruited. The behavior that is measured is entirely that of the initial bundle of recruited fibers, with no progressive recruitment of the remaining fibers due to the constant strain maintained throughout the test.

With the exception of the 10BY fibrous system, the composite structures stabilize under greater true stress intensities than the corresponding controls, as shown in Figure 9. At approximately 2 MPa, the 10BY composite stabilized, exhibiting the greatest tension loss (15.5 MPa). The 8BY composite achieved stability at a nominal level of approximately 2.5 MPa, albeit with a reduced stress drop of around 9.5 MPa. It can be deduced that the 6BY composite structure stabilizes under the greatest stress intensity (approximately 3.75 MPa) and experiences a stress drop of approximately 9.5 MPa, which is comparable to the stress drop observed in the PVA_8BY/10%HAp braided composite.

To summarize, similar to the creep tensile tests, the inclusion of HAp in the PVA_10BY/10%HAp system results in a diminished advantage. However, in the case of the PVA_6BY/10%HAp and PVA_8BY/10%HAp composites, their overall stress relaxation behavior remains essentially identical. It is important to note that there is a clear distinction between the relaxation and creep-characteristic responses in terms of the rate at which the equilibrium state is achieved. This is why the temporal characteristic of creep is commonly referred to as “retardation time”.

#### 3.3.8. DMA

**Figure 10:**
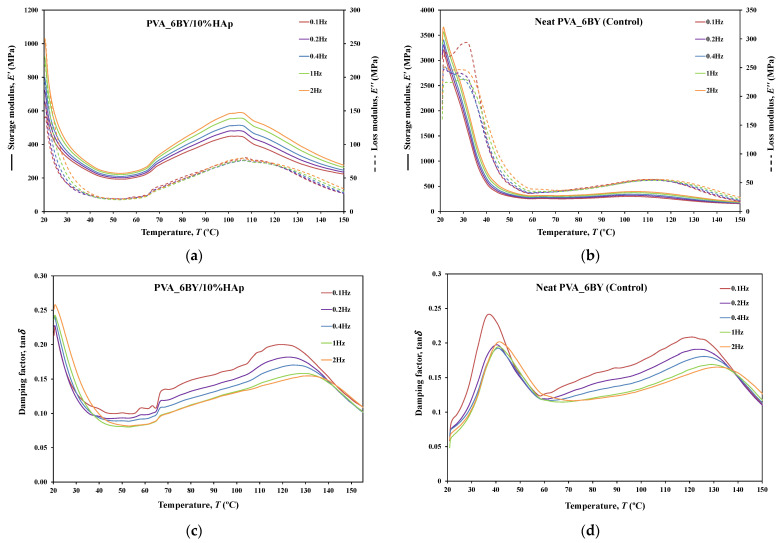
Tensile multi-frequency spectra of PVA_6BY/10%HAp and neat-PVA_6BY: (**a**,**b**) storage and loss moduli; (**c**,**d**) loss tangent.

**Figure 11:**
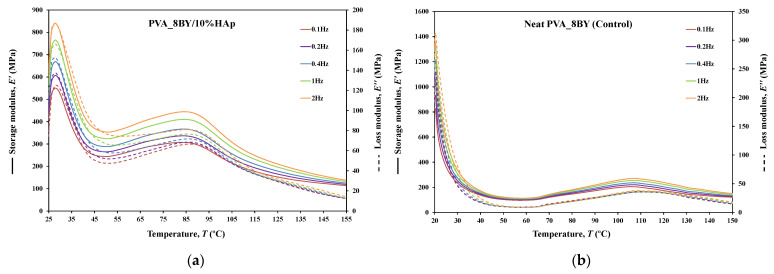

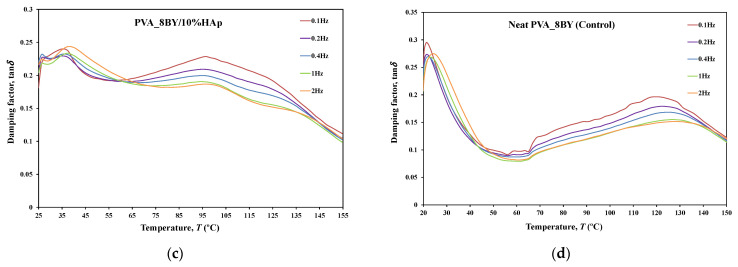
Tensile multi-frequency spectra of PVA_8BY/10%HAp and neat-PVA_8BY: (**a**,**b**) storage and loss moduli; (**c**,**d**) loss tangent.

**Figure 12:**
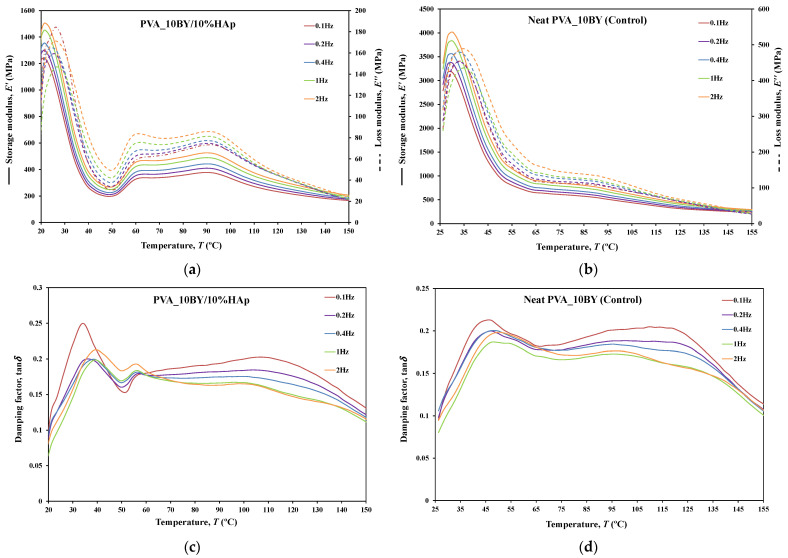
Tensile multi-frequency spectra of PVA_10BY/10%HAp and neat-PVA_10BY: (**a**,**b**) storage and loss moduli; (**c**,**d**) loss tangent.

The dynamic mechanical behavior of neat PVA and PVA/10%HAp composites was investigated in this study using DMA in tensile multi-frequency mode (0.1, 0.2, 0.4, 1, and 2 Hz). The storage ($E^{\prime }$) and loss ($E^{\prime \prime }$) moduli versus temperature curves, along with the loss tangent (tan*δ*) versus temperature curve, are shown in Figure 10, Figure 11 and Figure 12 for both neat-PVA braids and the matching PVA/10%HAp composite braided systems. By analyzing the increases and decreases of tan*δ*, its representation offers qualitative insights into the viscoelastic state of the material, given that it is derived from the ratio of loss to storage moduli. As a result, it permits the determination of the principal constituent (elastic or viscous) at a particular temperature and frequency.

The behavior of $E^{\prime }$ and $E^{\prime \prime }$ curves is comparable for every braided architecture that was tested (Figure 10a,b, Figure 11a,b and Figure 12a,b), except for the 10BY system. The curves representing PVA_6BY/10%HAp and PVA_8BY/10%HAp (illustrated in Figure 10a and Figure 11a) exhibit a very similar trend. This similarity can be outlined in three distinct regions, each of which corresponds to molecular motion and subsequent modulus variations. With respect to the first region, the PVA_6BY/10%HAp and PVA_8BY/10%HAp systems exhibit maximal peaks at approximately 20 °C and 28 °C, respectively, in both $E^{\prime }$ and $E^{\prime \prime }$. The second region is characterized by a lower and broader peak near 105 °C and 85 °C in the $E^{\prime }$ and $E^{\prime \prime }$ values, respectively, for PVA_6BY/10%HAp and PVA_8BY/10%HAp.

A diminishing frequency difference in the 8BY system is responsible for a constant decrease in moduli observed in the final region. However, the PVA_10BY/10%HAp braided composite exhibited a distinct pattern, featuring a third peak in addition to the two already observed at approximately 21 °C and 27 °C for $E^{\prime }$ and $E^{\prime \prime }$, respectively. Additionally, these findings indicate that the storage and loss moduli ($E^{\prime }$ and $E^{\prime \prime }$) increase with the loading frequency. Furthermore, it is consistently observed that the value of $E^{\prime }$ is four to ten times greater than the value of $E^{\prime \prime }$, albeit with an equivalent magnitude of difference.

Furthermore, the initial observation of a significant decrease in $E^{\prime }$ and $E^{\prime \prime }$ for all composite structures can be linked to the peaks at around 21C, 35C, and 35C in the tan*δ* of PVA_6BY/10%HAp, PVA_8BY/10%HAp, and PVA_10BY/10%HAp, respectively (depicted in Figure 10, Figure 11 and Figure 12c). The early peaks observed in the spectrum may be attributed to the beta relaxation ($T_{\beta }$), which is influenced by the glass transition temperature $T_{g}$ of the unhydrolyzed PVAc. Alternatively, they could arise from the movement of PVA side chain backbone segments that encompass acetate groups or crosslinking species. The PVA/10%HAp architectures demonstrate elastic behavior under tensile loading, as evidenced by the tan*δ* values ranging from 0.1 to 0.25 at physiological temperatures. This indicates that the composite systems possess minimal viscosity, which should not be disregarded. The tan*δ* curves for PVA_6BY/10%HAp and PVA_10BY/10%HAp (Figure 10c, Figure 11c and Figure 12c) exhibit a frequency-dependent behavior between 40–140 °C, whereas PVA_8BY/10%HAp demonstrates a more elastic response (lower value of tan*δ*) at high frequencies. This characteristic unequivocally identifies the existence of a glass transition point, denoted as $T_{g}$, within the specified temperature ranges.

Certainly, this event is characterized by a significant decrease in $E^{\prime }$ [62] (i.e., a reduction in stiffness, as in [23]), but it is predominantly denoted by a peak in $E^{\prime \prime }$, as it showcases the material’s capability to dissipate mechanical energy (i.e., it is linked to plastic response) [29,63,64]. The $T_{g}$ of a polymer plays a crucial role in comprehending the material’s effects on mechanical behavior, the level of crosslinking, and the alterations that occur within the bulk at a specific temperature [65]. This material parameter is of utmost importance. The crystallinity of the PVA polymer chains is a determining factor [23]. Therefore, the second and significantly wider peak is related to the $T_{g}$ of PVA, an alpha transition ($T_{\alpha }$) that characterizes the initial (primary) motion of the PVA main chain in the amorphous regions of the polymer matrix [45,66]. This means that $T_{g}$ values approximately equal to 107 °C, 89 °C, and 91 °C were identified for PVA_6BY/10%HAp, PVA_8BY/10%HAp, and PVA_10BY/10%HAp, respectively, being slightly different from those obtained in DTA. An increase in the $T_{g}$ values of composite systems (experimental group) was observed when compared to the corresponding neat PVA architectures (control group), except for the 8BY case, upon the incorporation of HAp. Similar outcomes were observed when HAp was incorporated into the polymeric matrix in a separate investigation [45]. A slight increase in the $T_{g}$ was observed, starting at 32 °C (representing pure PLGA) and reaching a maximum of 37 °C as the nano-HAp concentration increased from 0% to 10% (hindering the movement of the polymer chain) and then reaching a plateau. This phenomenon can be attributed to the strong interactions formed between PVA and HAp, which involve hydrogen bonding and [OH-]–Ca^2+^-[-OH] linkages. These interactions impose further constraints on the polymer chains, thereby limiting their segmental mobility [22,49]. The formation of such robust interactions could potentially result in unforeseeable fluctuations in the characteristics of the biomaterial [33]. Referring to the loss tangent (tan*δ*), it was observed that it continues to decrease at higher temperatures, up to 160 °C. However, the obtained response tends to converge to a frequency-independent value that is distinct, suggesting that the molecular orientation of the composite systems is increasing as a result of polymer stretching.

The composite structure PVA_10BY/10%HAp exhibits an additional peak. This behavior can be elucidated by the architectural and dimensional effects of the 10BY fibrous system, which are indicative of the 10BY composite system’s increased stiffness (as measured by higher $E^{\prime }$ values). The 10BY architecture, as illustrated in SEM Figure 2l, is coaxial (has a void in the center), which distinguishes it entirely from the architectures of 6BY and 8BY, which lack a central void.

As shown in Figure 10, Figure 11 and Figure 12c,d, the highest tan*δ* peak for neat PVA-braided fabrics occurs at 0.1 Hz, which corresponds to low damping or a high $E^{\prime }$. Moreover, in the absence of the 10BY system, the addition of HAp diminishes the peak intensity at the identical frequency value (0.1 Hz), indicating a greater decrease in damping or increase in $E^{\prime }$. The addition of HAp to neat PVA brains results in a decrease in their viscoelastic behavior, which subsequently shifts to an elastic response. The observation of this occurrence validates the brittleness of PVA/10%HAp composite braided in comparison to pure PVA structures [29], as the addition of HAp modifies the viscoelastic characteristics of the polymeric material. This is because the imposed restrictions on the mobility of the polymer chains reduce the damping (tan*δ*) [29] of braided composites consisting of PVA/10%HAp.

Furthermore, Figure 10, Figure 11 and Figure 12 illustrate that the incorporation of HAp leads to a reduction in $E^{\prime }$ (i.e., stiffness) of PVA braids, resulting in increased flexibility. Additionally, HAp exhibits a plasticizing influence, thereby improving the ductility of PVA-braided structures. The trend of these data is comparable to that of *E* as determined by uniaxial tensile measurements. Hence, the addition of HAp resulted in a marginal reduction in the stiffness of PVA braids, as demonstrated by both static and dynamic mechanical analysis.

## 4. Conclusions

This study presents an analysis of the morphological, chemical, thermal, and mechanical properties of PVA composite braids incorporated with 10%HAp. In order to achieve this objective, various techniques, including DMA investigations, SEM, EDS, TGA, DTA, FTIR, uniaxial monotonic tensile, creep, and relaxation tests, were conducted. Based on the obtained results, it can be concluded that the chosen method and procedure for adding HAp particles to the PVA braids were suitable and effective in the fabrication of the braided composite.

The surface of the fibers was coated with a dense and homogeneous dispersion of HAp crystals (i.e., a uniform HAp coating layer), as confirmed by SEM analysis. There were no significant agglomerates, which unquestionably increased the surface roughness of the PVA braids. The roughness of this composite material can be utilized as evidence for its bioactivity, as it facilitates enhanced osseointegration, which is the attachment of bone cells, accelerates cell proliferation, stimulates osteogenic differentiation, improves nutrient diffusion, and helps cell adhesion.

The EDS analysis revealed that the Ca/P atomic ratio was approximately 1.70, which is similar to the ratio found in natural bone (1.63 to 1.67). This finding provides confirmation that the inclusion of HAp results in PVA-braided architectures with remarkable bioactivity.

As a result of the addition of HAp, TGA analysis identified changes in thermal behavior. With the addition of HAp, the maximum loss mass (degradation) temperature decreased somewhat; however, the incorporation of HAp into the braids has no effect on the initial temperature of PVA decomposition.

The peaks of PVA/HAp composite braids exhibited a certain degree of similarity to the standard patterns of PVA and HAp, as determined by FTIR analysis.

Glass transition temperature ($T_{g}$) increased when HAp was incorporated into PVA braids, as demonstrated by DTA and DMA. This phenomenon is plausibly attributable to the robust interactions formed between PVA and HAp, including hydrogen bonding and [OH-]-Ca^2+^-[-OH] linkages, which impose additional restrictions on the polymer chains and impede their segmental mobility as a result.

The PVA/HAp composite braids showed superior mechanical properties, particularly in terms of tensile strength. Nevertheless, Young’s modulus (E) values of these composites fall short of those observed in human bone tissue. Although the specific cause for the comparatively lower E average values within the composite groups remains unclear, two potential explanations could be a stronger bond between the polymeric matrix and the filler particles, as well as a more cohesive response to the applied load by the microstructural components present in the PVA/10%HAp composite structures. DMA confirmed that the addition of HAp to PVA-braided architectures increased their ductility by plasticizing them. The observed data exhibit a comparable pattern to the E values obtained from uniaxial tensile measurements.

Based on creep and relaxation measurements, it was possible to conclude that the addition of HAp to the 8BY and 6BY composite architectures was advantageous. Moreover, in the case of the 10BY composite system, this effect fell completely away. As confirmed by SEM images, this behavior could be attributed to either its entirely distinct architecture or its higher HAp incorporation percentage.

This extensive analysis of PVA/HAp composites enables us to conclude that this reinforcement arrangement can be considered a potential biomaterial to be employed as a stabilizer of bone fractures, constituting capable solutions for further medical studies. These composites can be viewed as adequate solutions to be integrated into the production of fibrous fixation systems, thus offering adequate mechanical stability and biological compatibility that match the standard care for fracture healing, especially the multifragmentary ones. The production of biocomposite PVA/HAp braids is of interest both from fundamental science and applied perspectives.

## Figures and Tables

**Table 1:** Resume of the average thermal property values obtained through TG/DTG analyses for braided samples composed of pure PVA and PVA/10%HAp composite. CoV values (in percentage) are represented in curved parentheses.

Braided Architectures	*T* Peaks of DTG (°C) [Weight Loss, WL (%)]			Residual Weight at 500 °C (wt%)
	1st Thermal Degradation	2nd Thermal Degradation	3rd Thermal Degradation	
**Neat PVA_6BY**	80 (1) [6 (6)]	383 (1) [73 (1)]	448 (2) [16 (7)]	2 (9)
**Neat PVA_8BY**	80 (3) [6 (5)]	383 (0.3) [73 (1)]	447 (0.5) [16 (4)]	2 (12)
**Neat PVA_10BY**	82 (3) [6 (5)]	381 (0.4) [74 (1)]	446 (2) [16 (3)]	2 (38)
**PVA_6BY/10%HAp**	81 (3) [5 (9)]	379 (1) [66 (2)]	447 (1) [19 (5)]	7 (33)
**PVA_8BY/10%HAp**	79 (1) [5 (8)]	378 (1) [67 (2)]	450 (0.4) [19 (6)]	7 (24)
**PVA_10BY/10%HAp**	80 (2) [5 (4)]	374 (1) [64 (4)]	448 (0.9) [17 (12)]	12 (37)

**Table 2:** Thermal properties of neat PVA and PVA/10%HAp composite braided samples were analyzed using TG/DTG techniques, along with mean values and their corresponding coefficients of variation (in parenthesis).

Braided Architecture	Endothermal Peaks		
	1st	2nd	3rd
	*T*_g_ (°C)	*T*_m_ (°C)	PVA Degradation (°C)
**Neat-PVA_6BY**	68 (3)	216 (0.3)	366 (1)
**Neat-PVA_8BY**	68 (1)	216 (0.3)	366 (1)
**Neat-PVA_10BY**	72 (8)	215 (0.3)	356 (3)
**PVA_6BY/10%HAp**	77 (7)	215 (0.3)	353 (1)
**PVA_8BY/10%HAp**	72 (5)	215 (0.4)	346 (1)
**PVA_10BY/10%HAp**	74 (6)	215 (1)	344 (1)

**Table 3:** Mean values in quasi-static tensile loading tests (displacement rate of 100 mm.min^−1^) for neat PVA and PVA/10%HAp braids. Coefficient of variation (%) in parenthesis—CoV.

Braided Architecture	Number of Filaments/Fibres	Area, *A* (mm^2^)	Young’s Modulus, *E* (MPa)	Ultimate Tensile Strength *σ*^u^ (MPa)	Ultimate Strain, *ε*^u^ (%)
**Neat-PVA_6BY (Control)**	216	0.108	25.485 (3)	389.741 (1)	70.218 (0.1)
**Neat-PVA_8BY (Control)**	288	0.144	21.126 (10)	400.754 (5)	70.750 (3)
**Neat-PVA_10BY (Control)**	360	0.181	21.347 (12)	358.021 (8)	66.871 (9)
**PVA_6BY/10%HAp**	216	0.108	22.621 (9)	395.196 (5)	71.210 (3)
**PVA_8BY/10%HAp**	288	0.144	20.974 (10)	433.392 (5)	75.558 (3)
**PVA_10BY/10%HAp**	360	0.181	14.500 (16)	361.662 (12)	73.694 (6)

**Table 4:** Mean true strain within 7200 s for PVA-braided architectures of control and experimental groups ($\sigma_{0}=0.12\sigma^{u}$).

Braid Architecture	Applied Stress (MPa)	Strain (%)
**Neat-PVA_6BY (Control)**	46.77	ε=−6.9914203+5.7231542ln⁡(t)
**Neat-PVA_8BY (Control)**	48.09	ε=−7.6608032+5.6280758ln⁡(t)
**Neat-PVA_10BY (Control)**	42.96	ε=−7.6560895+6.0501519ln⁡(t)
**PVA_6BY/10%HAp**	47.42	ε=−5.3248416+5.1596638ln⁡(t)
**PVA_8BY/10%HAp**	52.01	ε=−4.8868559+4.6062576ln⁡(t)
**PVA_10BY/10%HAp**	43.40	ε=−9.6200959+7.2317279ln⁡(t)

**Table 5:** Mean true stress was obtained for the PVA-braided architectures of the control and experimental groups over a duration of 2700 s $\varepsilon_{0}=\varepsilon (0.12\sigma^{u})$.

Architecture	Applied Strain (%)	Stress (MPa)
Neat-PVA_6BY (Control)	2.3	σ=4.7693927−0.33327167ln(t)
Neat-PVA_8BY (Control)	2.0	σ=5.0096928−0.44016105ln(t)
Neat-PVA_10BY (Control)	2.5	σ=8.4988453−0.79249583ln(t)
PVA_6BY/10%HAp	2.6	σ=11.037776−0.92539288ln(t)
PVA_8BY/10%HAp	2.8	σ=9.6811132−0.92407862ln(t)
PVA_10BY/10%HAp	3.3	σ=14.260553−1.5792217ln(t)

## Data Availability

The raw/processed data required to reproduce these findings can be shared at this time, as the data are also part of an ongoing study.

## References

[1] Ratner B.D., Hoffman A.S., Schoen F.J., Lemons J.E. (2020). Biomaterials Science: An Introduction to Materials in Medicine.

[2] Stevens B., Yang Y., Mohandas A., Stucker B., Nguyen K.T. (2008). A review of materials, fabrication methods, and strategies used to enhance bone regeneration in engineered bone tissues. J. Biomed. Mater. Res. Part B Appl. Biomater..

[3] Fracture Healing Overview. https://www.ncbi.nlm.nih.gov/books/NBK551678/.

[4] Zhou K., He X., Tao X., Pan F., Yang H. (2020). A biomechanical matched-pair comparison of two different locking plates for tibial diaphyseal comminuted fracture: Carbon fiber-reinforced poly-ether-ether-ketone (CF-PEEK) versus titanium plates. J. Orthop. Surg. Res..

[5] Teixeira M.A., Amorim M.T.P., Felgueiras H.P. (2019). Poly(Vinyl Alcohol)-Based Nanofibrous Electrospun Scaffolds for Tissue Engineering Applications. Polymers.

[6] Marongiu G., Dolci A., Verona M., Capone A. (2020). The biology and treatment of acute long-bones diaphyseal fractures: Overview of the current options for bone healing enhancement. Bone Rep..

[7] Geiger M. (2003). Collagen sponges for bone regeneration with rhBMP-2. Adv. Drug Deliv. Rev..

[8] Radius and Ulnar Shaft Fractures. https://www.ncbi.nlm.nih.gov/books/NBK557681/.

[9] Rüedi T.P., Arraf J., Babst R., Balogh Z.J., Barbosa P., Barla J.D., Baumgaertel F., Bernstein B., Blauth M., Borens O., Buckley R.E., Moran C.G., Apivatthakakul T. (2018). Humerus, shaft. AO Principles of Fracture Management.

[10] Rüedi T.P., Arraf J., Babst R., Balogh Z.J., Barbosa P., Barla J.D., Baumgaertel F., Bernstein B., Blauth M., Borens O., Buckley R.E., Moran C.G., Apivatthakakul T. (2018). Forearm, shaft. AO Principles of Fracture Management.

[11] Rüedi T.P., Arraf J., Babst R., Balogh Z.J., Barbosa P., Barla J.D., Baumgaertel F., Bernstein B., Blauth M., Borens O., Buckley R.E., Moran C.G., Apivatthakakul T. (2018). Biology and biomechanics in bone healing. AO Principles of Fracture Management.

[12] Lia T.-T., Ling L., Lin M.-C., Jiang Q., Lin Q., Lou C.-W., Lin J.-H. (2019). Effects of ultrasonic treatment and current density on the properties of hydroxyapatite coating via electrodeposition and its in vitro biomineralization behavior. Mater. Sci. Eng. C.

[13] Reith G., Schmitz-Greven V., Hensel K.O., Schneider M.M., Tinschmann T., Bouillon B., Probst C. (2015). Metal implant removal: Benefits and drawbacks—A patient survey. BMC Surg..

[14] Rüedi T.P., Arraf J., Babst R., Balogh Z.J., Barbosa P., Barla J.D., Baumgaertel F., Bernstein B., Blauth M., Borens O., Buckley R.E., Moran C.G., Apivatthakakul T. (2018). Implants and biotechnology. AO Principles of Fracture Management.

[15] Perren S.M. (2002). Evolution of the internal fixation of long bone fractures: The scientific basis of biological internal fixation: Choosing a new balance between stability and biology. J. Bone Jt. Surg..

[16] Ozan S., Munir K., Biesiekierski A., Ipek R., Li Y., Wen C., Wagner W.R., Sakiyama-Elbert S., Zhang G., Yaszemski M. (2020). Titanium Alloys, Including Nitinol. Biomaterials Science: An Introduction to Materials in Medicine.

[17] Rüedi T.P., Arraf J., Babst R., Balogh Z.J., Barbosa P., Barla J.D., Baumgaertel F., Bernstein B., Blauth M., Borens O., Buckley R.E., Moran C.G., Apivatthakakul T. (2018). Tension band principle. AO Principles of Fracture Management.

[18] Li T.-T., Zhang Y., Ling L., Lin M.-C., Wang Y., Wu L., Lin J.-H., Lou C.-W. (2020). Manufacture and characteristics of HA-Electrodeposited polylactic acid/polyvinyl alcohol biodegradable braided scaffolds. J. Mech. Behav. Biomed. Mater..

[19] Lin J.-H., Lee M.-C., Chen C.-K., Huang C.-L., Chen Y.-S., Wen S.-P., Kuo S.-T., Lou C.-W. (2017). Recovery evaluation of rats’ damaged tibias: Implantation of core-shell structured bone scaffolds made using hollow braids and a freeze-thawing process. Mater. Sci. Eng. C.

[20] Qin Q., Liu Y., Chen S.-C., Zhai F.-Y., Jing X.-K., Wang Y.Z. (2012). Electrospinning fabrication and characterization of poly(vinyl alcohol)/layered double hydroxides composite fibers. J. Appl. Polym. Sci..

[21] Poursamar S.A., Rabiee M., Samadikuchaksaraei A., Tahriri M., Karimi M., Azami M. (2009). Influence of the value of the pH on the preparation of nano hydroxyapatite—Poly vinyl alcohol composites. J. Ceram. Process. Res..

[22] Enayati M.S., Behzad T., Sajkiewicz P., Bagheri R., Ghasemi-Mobarakeh L., Kuśnieruk S., Rogowska-Tylman J., Pahlevanneshan Z., Choińska E., Święszkowski W. (2016). Fabrication and characterization of electrospun bionanocomposites of poly (vinyl alcohol)/nanohydroxyapatite/cellulose nanofibers. Int. J. Polym. Mater. Polym..

[23] Jain N., Ali S., Singh V.K., Singh K., Bisht N., Chauhan S. (2019). Creep and dynamic mechanical behavior of cross-linked polyvinyl alcohol reinforced with cotton fiber laminate composites. J. Polym. Eng..

[24] Li S., Li G., Hu J., Wang B. (2021). Porous polyetheretherketone-hydroxyapatite composite: A candidate material for orthopedic implant. Compos. Commun..

[25] Li T.-T., Ling L., Lin M.-C., Jiang Q., Lin Q., Lin J.-H., Lou C.-W. (2019). Properties and Mechanism of Hydroxyapatite Coating Prepared by Electrodeposition on a Braid for Biodegradable Bone Scaffolds. Nanomaterials.

[26] Freire T.F., Quinaz T., Fertuzinhos A., Nguyễn T.Q., Moura M.F.S.M., Martins M., Zille A., Dourado N. (2021). Thermal, Mechanical and Chemical Analysis of Poly(vinyl alcohol) Multifilament and Braided Yarns. Polymers.

[27] Xiong D. (2010). Preparation and characterization of nano-hydroxyapatite/polyvinyl alcohol gel composites. J. Wuhan Univ. Technol. Sci. Ed..

[28] Wu G., Su B., Zhang W., Wang C. (2008). In vitro behaviors of hydroxyapatite reinforced polyvinyl alcohol hydrogel composite. Mater. Chem. Phys..

[29] Enayati M.S., Neisiany R.E., Sajkiewicz P., Behzad T., Denis P., Pierini F. (2018). Effect of nanofiller incorporation on thermomechanical and toughness of poly (vinyl alcohol)-based electrospun nanofibrous bionanocomposites. Theor. Appl. Fract. Mech..

[30] Uma Maheshwari S., Samuel V.K., Nagiah N. (2014). Fabrication and evaluation of (PVA/HAp/PCL) bilayer composites as potential scaffolds for bone tissue regeneration application. Ceram. Int..

[31] Pearce H.A., Kim Y.S., Diaz-Gomez L., Mikos A.G., Wagner W.R., Sakiyama-Elbert S., Zhang G., Yaszemski M. (2020). Tissue Engineering Scaffolds. Biomaterials Science: An Introduction to Materials in Medicine.

[32] Gibson I.R., Wagner W.R., Sakiyama-Elbert S., Zhang G., Yaszemski M. (2020). Natural and Synthetic Hydroxyapatites. Biomaterials Science: An Introduction to Materials in Medicine.

[33] Enayati M.S., Behzad T., Sajkiewicz P., Bagheri R., Ghasemi-Mobarakeh L., Łojkowski W., Pahlevanneshan Z., Ahmadi M. (2016). Crystallinity study of electrospun poly (vinyl alcohol) nanofibers: Effect of electrospinning, filler incorporation, and heat treatment. Iran. Polym. J..

[34] Kumar A., Negi Y.S., Choudhary V., Bhardwaj N.K. (2014). Microstructural and mechanical properties of porous biocomposite scaffolds based on polyvinyl alcohol, nano-hydroxyapatite and cellulose nanocrystals. Cellulose.

[35] Ruiz-Santos R., Monreal-Romero H., Chacon-Nava J.G. (2017). PVA/HAp composite with pork bone precursor obtained by electrospinning. Micro Nano Lett..

[36] Rodrigues P.J.G., Elias C.M.V., Viana B.C., Hollanda L.M., Stocco T.D., Vasconcellos L.M.R., Mello D.C.R., Santo F.E.P., Marciano F.R., Lobo A.O. (2020). Electrodeposition of bactericidal and bioactive nano-hydroxyapatite onto electrospun piezoelectric polyvinylidene fluoride scaffolds. J. Mater. Res..

[37] Li T.-T., Zhang Y., Ren H.-T., Peng H.-K., Lou C.-W., Lin J.-H. (2021). Two-step strategy for constructing hierarchical pore structured chitosan–hydroxyapatite composite scaffolds for bone tissue engineering. Carbohydr. Polym..

[38] Wiria F.E., Chua C.K., Leong K.F., Quah Z.Y., Chandrasekaran M., Lee M.W. (2008). Improved biocomposite development of poly(vinyl alcohol) and hydroxyapatite for tissue engineering scaffold fabrication using selective laser sintering. J. Mater. Sci. Mater. Med..

[39] Shah D.S., Lawson B.K., Yaszemski M., Wagner W.R., Sakiyama-Elbert S., Zhang G., Yaszemski M. (2020). Description and Definition of Adhesives, and Related Terminology. Biomaterials Science: An Introduction to Materials in Medicine.

[40] Hussain R., Tabassum S., Gilani M.A., Ahmed E., Sharif A., Manzoor F., Shah A.T., Asif A., Sharif F., Iqbal F. (2016). In situ synthesis of mesoporous polyvinyl alcohol/hydroxyapatite composites for better biomedical coating adhesion. Appl. Surf. Sci..

[41] Buckley R.E. (2013). Locking plates: A current concepts review of technique and indications for use. Acta Chir. Orthop. Traumatol. Cech..

[42] Rüedi T.P., Arraf J., Babst R., Balogh Z.J., Barbosa P., Barla J.D., Baumgaertel F., Bernstein B., Blauth M., Borens O., Buckley R.E., Moran C.G., Apivatthakakul T. (2018). External fixator. AO Principles of Fracture Management.

[43] Regi M.V., Esbrit P., Salinas A.J., Wagner W.R., Sakiyama-Elbert S., Zhang G., Yaszemski M. (2020). Degradative Effects of the Biological Environment on Ceramic Biomaterials. Biomaterials Science: An Introduction to Materials in Medicine.

[44] Laurencin C.T., Wagner W.R., Sakiyama-Elbert S., Zhang G., Yaszemski M. (2020). Bone Tissue Engineering. Biomaterials Science: An Introduction to Materials in Medicine.

[45] Jose M., Thomas V., Johson K., Dean D., Nyairo E. (2009). Aligned PLGA/HA nanofibrous nanocomposite scaffolds for bone tissue engineering. Acta Biomater..

[46] Chocholata P., Kulda V., Dvorakova J., Kolaja Dobra J., Babuska V. (2020). Biological Evaluation of Polyvinyl Alcohol Hydrogels Enriched by Hyaluronic Acid and Hydroxyapatite. Int. J. Mol. Sci..

[47] Chen F., Wang Z.-C., Lin C.-J. (2002). Preparation and characterization of nano-sized hydroxyapatite particles and hydroxyapatite/chitosan nano-composite for use in biomedical materials. Mater. Lett..

[48] Zeng S., Fu S., Guo G., Liang H., Qian Z., Tang X., Luo F. (2011). Preparation and Characterization of Nano-Hydroxyapatite/Poly(vinyl alcohol) Composite Membranes for Guided Bone Regeneration. J. Biomed. Nanotechnol..

[49] Asran A.S., Henning S., Michler G.H. (2010). Polyvinyl alcohol–collagen–hydroxyapatite biocomposite nanofibrous scaffold: Mimicking the key features of natural bone at the nanoscale level. Polymer.

[50] El-aziz A.M.A., El-Maghraby A., Taha N.A. (2017). Comparison between polyvinyl alcohol (PVA) nanofiber and polyvinyl alcohol (PVA) nanofiber/hydroxyapatite (HA) for removal of Zn^2+^ ions from wastewater. Arab. J. Chem..

[51] Yang C.-C., Lin C.-T., Chiu S.-J. (2008). Preparation of the PVA/HAP composite polymer membrane for alkaline DMFC application. Desalination.

[52] Satpathy A., Pal A., Sengupta S., Das A., Hasan M.M., Ratha I., Barui A., Bodhak S. (2019). Bioactive Nano-Hydroxyapatite Doped Electrospun PVA-Chitosan Composite Nanofibers for Bone Tissue Engineering Applications. J. Indian Inst. Sci..

[53] Mollazadeh S., Javadpour J., Khavandi A. (2007). Biomimetic synthesis and mechanical properties of hydroxyapatite/poly (vinyl alcohol) nanocomposites. Adv. Appl. Ceram..

[54] Koski A., Yim K., Shivkumar S. (2004). Effect of molecular weight on fibrous PVA produced by electrospinning. Mater. Lett..

[55] Lou C.W., Kuo S.T., Wen S.P., Lin J.H. (2014). Braided Bone Scaffolds Made by Braiding Polyvinyl Alcohol and Cross-Linked by Glutaraldehyde: Manufacturing Process and Structure Evaluation. Adv. Mater. Res..

[56] Jamil N., Husin H., Alfida A.W., Aman Z., Hassan Z. (2018). Characterization and Preparation of Polyvinyl Alcohol (PVA) as Inhibitor in Formation of Hydrates. Int. J. Curr. Res. Sci. Eng. Technol..

[57] Jia X., Li Y., Cheng Q., Zhang S., Zhang B. (2007). Preparation and properties of poly(vinyl alcohol)/silica nanocomposites derived from copolymerization of vinyl silica nanoparticles and vinyl acetate. Eur. Polym. J..

[58] Campa-Siqueiros P., Madera-Santana T.J., Ayala-Zavala J.F., López-Cervantes J., Castillo-Ortega M.M., Herrera-Franco P.J. (2020). Nanofibers of gelatin and polivinyl-alcohol-chitosan for wound dressing application: Fabrication and characterization. Polímeros.

[59] Cooper J.A., Lu H.H., Ko F.K., Freeman J.W., Laurencin C.T. (2005). Fiber-based tissue-engineered scaffold for ligament replacement: Design considerations and in vitro evaluation. Biomaterials.

[60] Prakash J., Kumar T.S., Venkataprasanna K.S., Niranjan R., Kaushik M., Samal D.B., Venkatasubbu G.D. (2019). PVA/alginate/hydroxyapatite films for controlled release of amoxicillin for the treatment of periodontal defects. Appl. Surf. Sci..

[61] Thornton G.M., Frank C.B., Shrive N.G. (2001). Ligament creep behavior can be predicted from stress relaxation by incorporating fiber recruitment. J. Rheol..

[62] Moraes M.R., Alves A.C., Toptan F., Martins S.C., Vieira E.M.F., Paleo A.J., Souto A.P., Santos W.L.F., Estevesa M.F., Zille A. (2017). Glycerol/PEDOT:PSS coated woven fabric as a flexible heating element on textiles. J. Mater. Chem. C.

[63] Menard K.P. (2008). Dynamic Mechanical Analysis.

[64] Wilcox A.G., Buchan K.G., Espino D.M. (2014). Frequency and diameter dependent viscoelastic properties of mitral valve chordae tendineae. J. Mech. Behav. Biomed. Mater..

[65] Aldred N., Wills T., Williams D., Clare A. (2007). Tensile and dynamic mechanical analysis of the distal portion of mussel (*Mytilus edulis*) byssal threads. J. R. Soc. Interface.

[66] Jain N., Singh V.K., Chauhan S. (2018). Dynamic and creep analysis of polyvinyl alcohol based films blended with starch and protein. J. Polym. Eng..

